# The LINC00961 transcript and its encoded micropeptide, small regulatory polypeptide of amino acid response, regulate endothelial cell function

**DOI:** 10.1093/cvr/cvaa008

**Published:** 2020-01-28

**Authors:** Helen L Spencer, Rachel Sanders, Mounia Boulberdaa, Marco Meloni, Amy Cochrane, Ana-Mishel Spiroski, Joanne Mountford, Costanza Emanueli, Andrea Caporali, Mairi Brittan, Julie Rodor, Andrew H Baker

**Affiliations:** 1 University/BHF Centre for Cardiovascular Science, Queens Medical Research Institute, University of Edinburgh, 47 Little France Crescent, Edinburgh EH16 4TJ, UK; 2 Institute of Cardiovascular and Medical Sciences, University of Glasgow, 126 University Pl, Glasgow G12 8TA, UK; 3 National Heart and Lung Institute, Vascular Sciences and Cardiac Function, Imperial Centre for Translational and Experimental Medicine, Imperial College London, London W12 0NN, UK

**Keywords:** Angiogenesis, LncRNA, Endothelial cell, Micropeptide, Hind limb ischaemia

## Abstract

**Aims:**

Long non-coding RNAs (lncRNAs) play functional roles in physiology and disease, yet understanding of their contribution to endothelial cell (EC) function is incomplete. We identified lncRNAs regulated during EC differentiation and investigated the role of *LINC00961* and its encoded micropeptide, small regulatory polypeptide of amino acid response (SPAAR), in EC function.

**Methods and results:**

Deep sequencing of human embryonic stem cell differentiation to ECs was combined with Encyclopedia of DNA Elements (ENCODE) RNA-seq data from vascular cells, identifying 278 endothelial enriched genes, including 6 lncRNAs. Expression of LINC00961, first annotated as an lncRNA but reassigned as a protein-coding gene for the SPAAR micropeptide, was increased during the differentiation and was EC enriched. *LINC00961* transcript depletion significantly reduced EC adhesion, tube formation, migration, proliferation, and barrier integrity in primary ECs. Overexpression of the SPAAR open reading frame increased tubule formation; however, overexpression of the full-length transcript did not, despite production of SPAAR. Furthermore, overexpression of an ATG mutant of the full-length transcript reduced network formation, suggesting a bona fide non-coding RNA function of the transcript with opposing effects to SPAAR. As the LINC00961 locus is conserved in mouse, we generated an LINC00961 locus knockout (KO) mouse that underwent hind limb ischaemia (HLI) to investigate the angiogenic role of this locus *in vivo*. In agreement with *in vitro* data, KO animals had a reduced capillary density in the ischaemic adductor muscle after 7 days. Finally, to characterize *LINC00961* and SPAAR independent functions in ECs, we performed pull-downs of both molecules and identified protein-binding partners. *LINC00961* RNA binds the G-actin sequestering protein thymosin beta-4x (Tβ4) and Tβ4 depletion phenocopied the overexpression of the ATG mutant. SPAAR binding partners included the actin-binding protein, SYNE1.

**Conclusion:**

The LINC00961 locus regulates EC function *in vitro* and *in vivo*. The gene produces two molecules with opposing effects on angiogenesis: SPAAR and *LINC00961*.

## Graphical Abstract

**Graphical Abstract:**
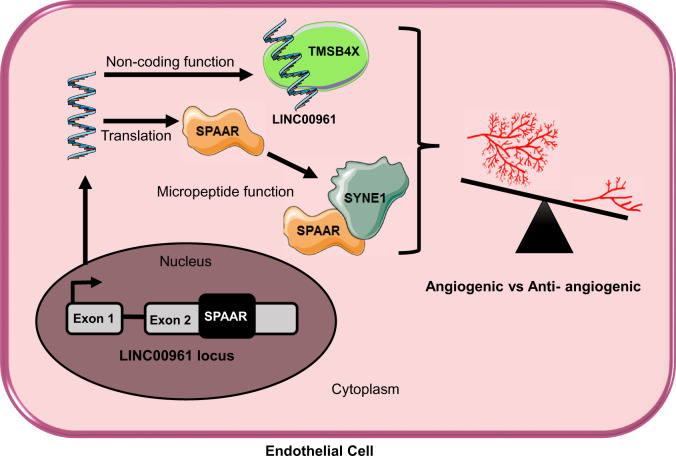

## 1. Introduction

The endothelium is a heterogeneous organ system that regulates homeostasis of the vasculature and represents a permeable monolayer barrier between the vessel wall and the blood. Endothelial cells regulate and adapt to shear stress, leucocyte extravasation, blood clotting, inflammation, vascular tone, extracellular matrix deposition, vasoconstriction/vasodilation, and angiogenesis. During angiogenesis, ECs become activated and undergo sprouting, proliferation, migration along a gradient of pro-angiogenic factors [e.g. vascular endothelial growth factor (VEGF), fibroblast growth factor (FGF), and platelet-derived growth factor (PDGF)], and anastomose to form new capillaries before returning to their quiescent state.^1^ Aberrant activation however, leads to EC dysfunction that can cause systemic vascular pathology.^1^^,^^2^ This uncontrolled activation is a significant factor contributing to coronary artery disease, diabetes, hypertension patients, hypercholesterolaemia, lupus, and has been reported as increased in smokers.^3^^,^^4^

Several groups have demonstrated the ability to differentiate ECs from human embryonic stem cells (hESC).^5–7^ This protocol yields ECs that are relatively immature and express genes that are somewhat distinct from those of mature ECs from various vascular beds,^8^ highlighting the importance of understanding the molecular mechanisms controlling both general and specialized EC differentiation, specification, and function. These derived ECs have been extensively proven to be functional both *in vitro*, by the ability to form capillary-like networks on Matrigel^7^ and *in vivo*, by their ability to improve vascular density and perfusion in a murine model of hind limb ischaemia (HLI).^9^ These data provide evidence of the benefits to hESC-derived EC for therapeutics and as a model to characterize early vascular development.

Data from the human Encyclopedia of DNA Elements (ENCODE) project indicate that approximately 93% of the genome is transcribed, with less than 2% encoding protein sequences.^10^ Currently, these non-coding RNAs (ncRNAs) are classified based on size, into long non-coding RNAs (lncRNAs) >200 bp and small ncRNAs <200 bp. LncRNAs correspond to a heterogeneous class of genes, with subtypes classified based on neighbouring protein-coding genes. In particular, lincRNAs are intergenic lncRNAs with no overlap with protein-coding genes. While some lincRNAs regulate in *cis* their protein-coding neighbours expression, a large range of *trans*-functions have been reported including chromatin remodelling, transcriptional and post-transcriptional regulation, translation control, and regulation of protein activity.^11^ LncRNAs show spatio-temporal expression, and are poorly conserved between species^12^; however, to date only a few of the lncRNAs known to exist have been functionally characterized. Recent literature highlights the important functions of lncRNAs as regulators of the cardiovascular system.^13^^,^^14^ In the vascular endothelium, TIE1-AS1 was the first described endothelial-specific lncRNA, involved in modulating TIE-1 expression and regulating endothelial vessel formation.^15^ A comprehensive transcriptome analysis of early cardiovascular development revealed the regulation of several lncRNAs and led to the characterization of ALIEN and PUNISHER.^16^ Recently, the hypoxia-induced lncRNA, GATA6-AS, was shown to epigenetically regulate angiogenesis through its interaction with the epigenetic regulator LOXL2.^17^^,^^18^

The ‘non-coding’ property of some lncRNAs has been disputed by the discovery of small open reading frames (ORFs) in some lncRNA transcripts, able to generate functional micropeptides.^19^^,^^20^ For example, LINC00948 has been reclassified as a protein-coding gene, as it encodes myoregulin, which inhibits the calcium ATPase SERCA in muscle.^21^ Similarly, the micropeptide DWORF encoded by lncRNA NONMMUG026737 activates the SERCA pump.^22^ Noteworthy to this study, a conserved micropeptide termed small regulatory polypeptide of amino acid response (SPAAR) was recently shown to be encoded by the *LINC00961* locus.^23^ SPAAR attenuates lysosomal v-ATPases interaction with mTORC1 under amino acid stimulation and modulates skeletal muscle regeneration following cardiotoxin injury.^23^ These studies focused on the function of the derived micropeptide; however, some micropeptides have been shown to be expressed from lncRNAs with previously characterized non-coding functions,^24^ suggesting the possibility of bi-functional loci.

We identified the *LINC00961*/SPAAR locus as EC enriched and sought to identify the role of this micropeptide-encoding gene. This led to dissection of the contribution of the *LINC00961* RNA transcript itself and the SPAAR micropeptide on endothelial function. *LINC00961* RNA was found to act as a bona fide lncRNA that inhibited angiogenesis and bound to the known angiogenic and actin-binding protein thymosin beta 4-x (Tβ4). Whereas SPAAR was found to be pro-angiogenic and bound to another actin-binding protein, SYNE1.

## 2. Methods

### 2.1 EC isolation and cell culture

All donated tissues have been obtained under proper informed consent and the investigation conforms with principles in the Declaration of Helsinki. Human saphenous vein endothelial cells (HSVECs) were obtained by enzymatic collagenase digestion of human saphenous veins (Ethics 15/ES/0094). Human umbilical vein endothelial cells (HUVEC) were obtained from Lonza (Basel Switzerland).

### 2.2 RNA-Seq of hESC differentiation to ECs

A previously published protocol was employed to generate ECs from H9 hESCs.^7^ RNA-Seq analysis was performed as previously described^25^ with minor modifications. Ensembl GRCh38 was used for transcriptome annotation. Read counts for each gene were obtained using HTSeq.^26^ The differential expression was analysed using DESeq2.^27^ RNA-seq data are deposited at the Gene expression Omnibus as GSE118106.

Expression data from several human endothelial and smooth muscle cell (SMC) lines were obtained from the ENCODE Consortium. The list of analysed data and their abbreviated name can be found in Supplementary material online, *Table S1*. Candidate filtering was done as follow: (i) Genes enriched in day 7 EC vs. hESC and non-EC day sample based on a LogFC ≥ 1, *P*adj < 0.01, FPKM ≥ 2; (ii) Genes up-regulated in HSVEC vs. hESC (LogFC ≥ 1, *P*adj < 0.01, FPKM ≥ 2); (iii) Genes expressed in ENCODE ECs (min of 2 FPKM in 10 samples); (iv) Enriched expressed in ENCODE ECs vs. ENCODE SMCs (two-fold enrichment between the average expression in ECs and SMCs).

### 2.3 HUVEC transfection and phenotype analysis

All phenotypes were assessed in HUVECs at 24 h after transfection with dicer substrate siRNA (dsiRNA) or infection with lentiviral constructs (details of reagents and protocols in Supplementary material online, *Methods*). *In vitro* tubule network formation was assessed using Matrigel (Corning, USA) according to the manufacturer’s protocol. Proliferation was assessed using the Click-it EdU 488 Proliferation assay (Life Technologies, UK). Migration and endothelial barrier function assays were performed using an Electric Cell-substrate Impedance Sensing (ECIS) machine (Applied BioPhysics, USA) and cell viability assessed with a FITC Annexin V Detection Kit with PI (BioLegend).

### 2.4 Hind limb ischaemia

All animal experiments were performed in accordance with the Animals (Scientific Procedures) Act (UK) 1986 and under the auspices of UK Home Office Project and Personal Licenses held within The University of Edinburgh facilities. LINC00961^−/−^mouse line was obtained from Taconic©. Validation of genotype was two-fold. Ear clip samples from pups were sent to Transnetyx© for genotyping, and in-house validation was also carried out using qRT-PCR on mRNA extracted from the kidney. Surgical procedures were performed under inhaled general anaesthesia (isoflurane at 5% for induction and 1–2% for maintenance) and with appropriate peri-operative analgesic cover (subcutaneous injection of buprenorphine at 0.05 mg/kg). Unilateral HLI was surgically induced by left femoral artery ligation at two points and cauterization of this segment of artery, leaving the femoral vein and nerve untouched. Mice were maintained for 7 days after surgery. Male LINC00961^−/−^and wild type (WT) littermates on the C57Bl/b6NTAC were studied at 11 weeks of age. Animals were euthanized with pentobarbital (160 mg/kg) given by intraperitoneal injection. Tissues were perfusion fixed with PBS at 6 mL/min with a micropump and then with 4% paraformaldehyde at 6 mL/min.

### 2.5 Pull-down

*LINC00961* RNA pull-down was carried out with 50 pmol biotinylated lncRNA, obtained using the T7 RiboMAX Express Large Scale RNA Production System (Promega, UK). The biotinylated lncRNA was incubated with streptavidin magnetic beads and 20 µg of HUVECs protein lysate, using the Pierce Mag RNA Protein Pull-down kit (Thermo Scientific). For the SPAAR pull-down HUVECs expressing either LV-Null, LV-SPAAR untagged, or LV-SPAAR-HA tagged were cultured in EGM-2 media. Immunoprecipitation with either anti-IgG or anti-HA antibody was performed in two replicates. SPAAR binding partners were defined as proteins detected in the two pull-down replicates and with a two-fold enrichment compared with the IgG pull-down controls or pull-down in cells not overexpressing HA-tagged SPAAR. Keratin contaminants and unknown proteins were removed from the final candidate list.

### 2.6 Statistical analysis

Statistical analysis was performed as described in the figure legends using GraphPad Prism version 5.0. Data are expressed as mean ± SEM. Comparisons between two groups were analysed using two-tailed unpaired Student’s *t*-tests. Comparisons between more than two groups were analysed using one-way ANOVA. For qRT-PCR analysis, graphs display the expression relative to the housekeeping gene based on the double dCt analysis while the statistical analyses were done on dCt values. For data represented as fold change, the statistical analysis was done on the Log2 fold change using an one sample *t*-test.

## 3. Results

### 3.1 Identification of EC-enriched genes

**Figure 1:**
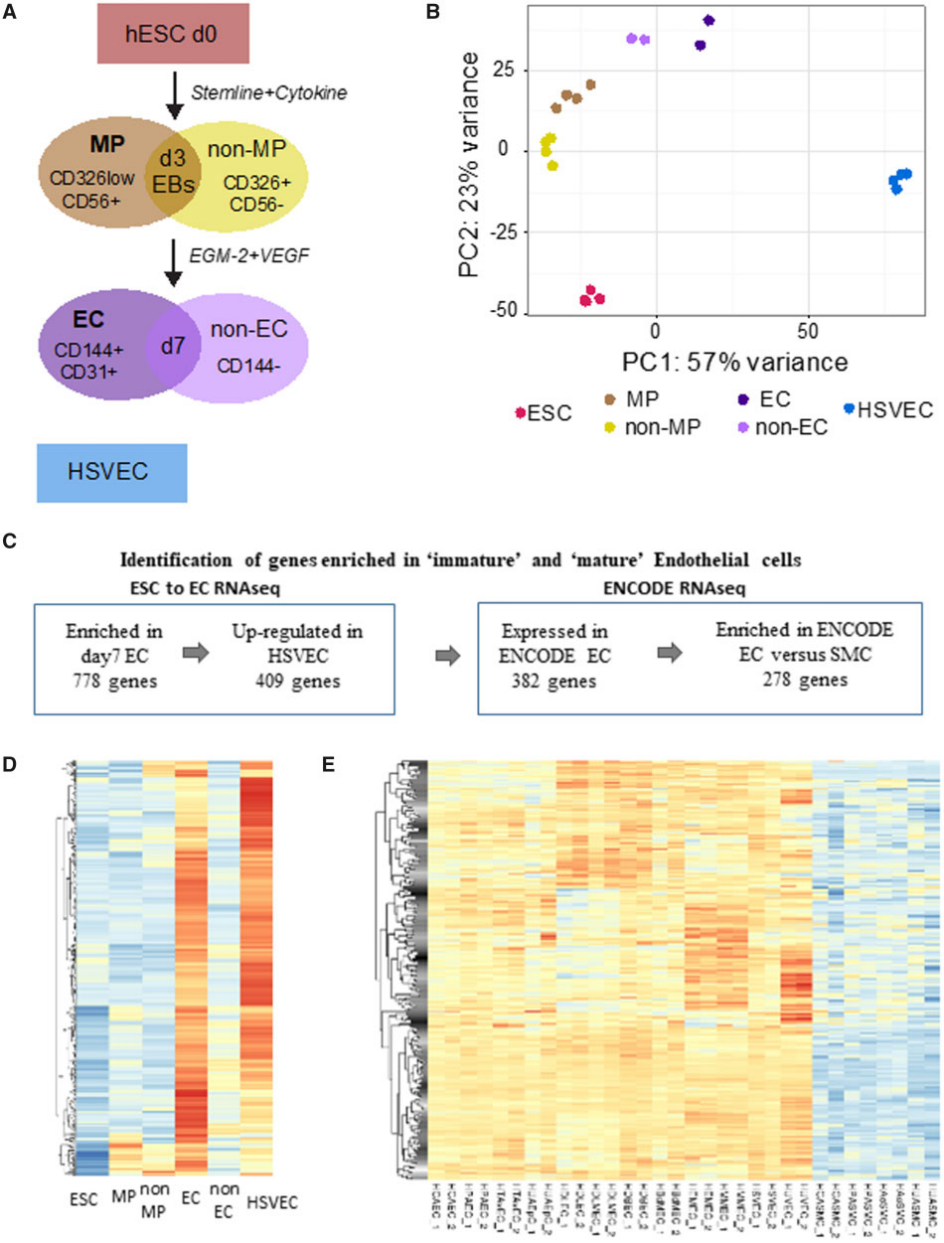
Identification of endothelial cell enriched genes. (*A*) Schematic representation of the RNA-seq samples: day 0 H9 hESC (ESC); Day 3 mesodermal population CD326^low^CD56^+^ (MP); Day 3 remaining population (non-MP); Day 7 EC CD144^+^CD31^+^(EC); Day 7 remaining population (non-EC); HSVEC. (*B*) PCA of the RNA-seq samples. The plot was generated on the regularized log-transformed data using DESEq2. (*C*) Summary of the selection of candidates to identify genes enriched in ‘immature’ and ‘mature’ ECs. (*D*) Heatmap showing the expression data [as row z-score of the Log2(FPKM + 1)] during differentiation of the 278 EC-enriched genes. (*E*) Heatmap showing the expression data [as row z-score of the Log2 (FPKM + 1)] of the 278 EC-enriched genes in ENCODE RNA-seq samples.

To identify genes specifically induced during endothelial fate specification and differentiation, we utilized an embryoid body-based protocol to generate ECs from hESCs (*Figure 1A*).^7^ This protocol was previously shown to generate functional hESC-EC, expressing CD144 and CD31 and able to form tube-like structures on Matrigel.^7^ RNA-seq was performed (45 million paired-end reads per sample) on ribosomal RNA depleted libraries from several replicates of the different cell populations (*Figure 1A*). Principal component analysis (PCA) demonstrated tight clustering of replicates and segregation of populations (*Figure 1B*). The purified EC samples obtained at day 7 (d7 EC) were closer to the human saphenous vein EC (HSVEC) samples in the PCA plot, but clearly clustered separately suggesting the immaturity of this EC population (*Figure 1B*). As expected, hESC pluripotency markers showed a down-regulation after day 3 of differentiation while mesoderm markers are up-regulated. We confirmed the expression of several endothelial markers in the d7 EC population but also showed the expression of arterial, venous, and lymphatic phenotype markers, suggesting endothelial heterogeneity (Supplementary material online, *Figure S1A*). As expected, we observed a high overlap between the genes up-regulated in d7 EC vs. hESC and the genes up-regulated in HSVEC vs. hESC (Supplementary material online, *Figure S1B*), validating their endothelial identity. 

To identify genes important for endothelial identity and function, we focused on candidates showing high expression in immature and mature ECs. We specifically selected 409 genes enriched in the day7 EC population but also expressed in our HSVEC samples. Then, we took advantage of RNA-seq data from the ENCODE consortium to assess their expression in several EC lines from different origins but also in SMCs. We retrieved a list of 278 genes with high expression in ECs and lower expression in SMCs (Supplementary material online, *Table S2*). This list contains known markers of ECs including PECAM1, CDH5, and ERG, and the Gene Ontology (GO) analysis revealed the enrichment of terms related to vessel development and angiogenesis (Supplementary material online, *Figure S1C*).

### 3.2 LINC00961 is enriched in immature and mature ECs

**Figure 2:**
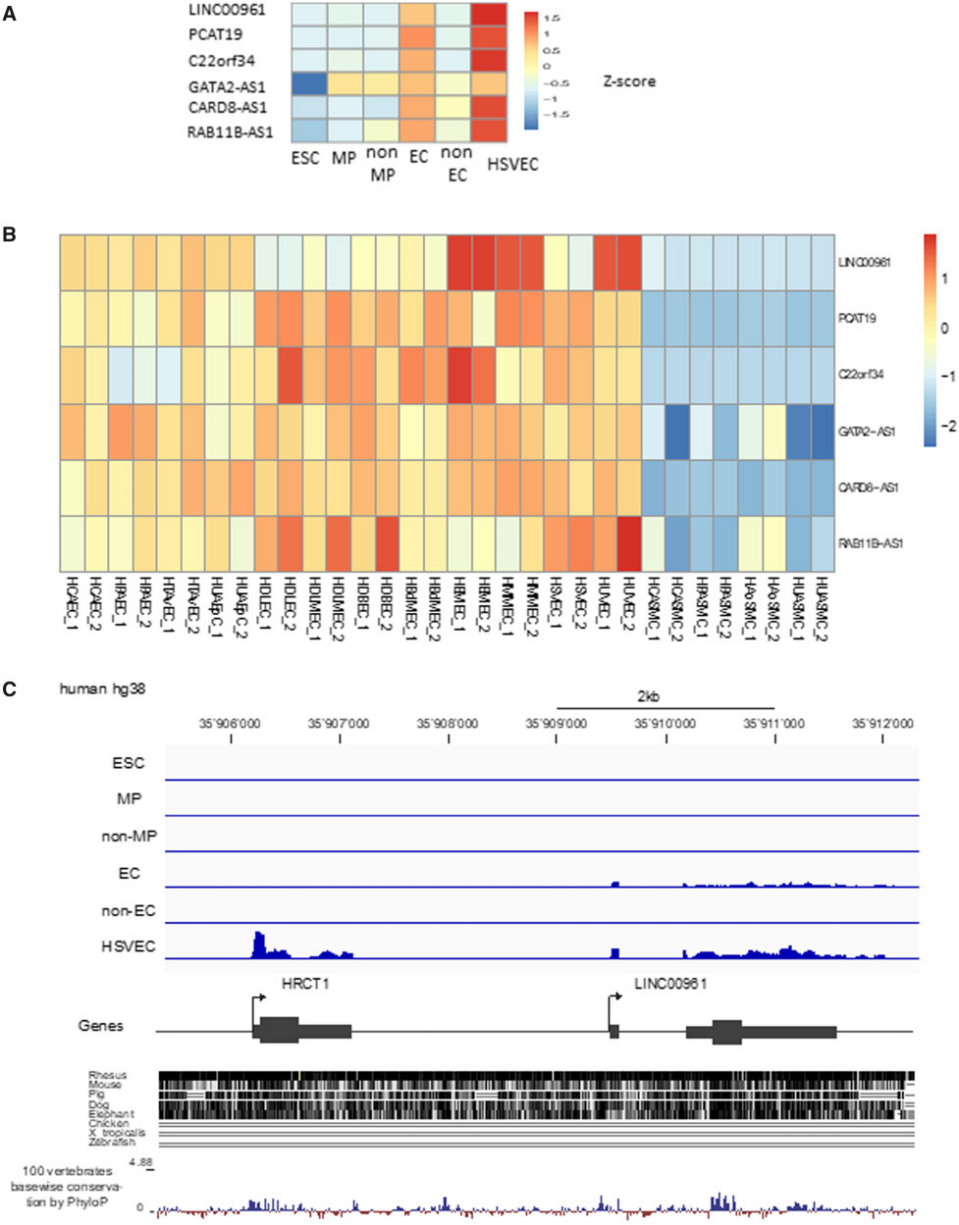
LINC00961 is enriched in immature and mature ECs. (*A*) Heatmap of the six lncRNAs identified in our EC differentiation protocol in each of the isolated cell populations. (*B*) Heatmap of these six lncRNAs in ENCODE RNA-seq samples including various types of EC lineages such as, venous, arterial, and lympthatic ECs. (*C*) Genomic organization of the LINC00961 gene, read profile from the ESC to EC RNA-seq, and conservation track based on UCSC alignment and PhyloP score.

Among the 278 genes enriched in immature and mature ECs, we found 6 lncRNAs: 3 antisense lncRNAs and 3 intergenic lncRNAs (*Figure 2A*, *B*). While antisense RNAs often regulate the expression of their sense genes,^24^ intergenic lncRNAs have function generally unrelated to their neighbouring protein-coding genes. From the three intergenic lincRNA, LINC00961 is the only one conserved in mouse (*Figure 2C*)*.* LINC00961 is located on chromosome 9 and while *LINC00961* transcript expression was detected in the d7 EC population and HSVECs with a read profile confirming a two-exon gene structure, neighbouring *HRCT1* expression was restricted to HSVECs (*Figure 2C*). Although LINC00961 was initially annotated as a lncRNA, the locus encodes a small ORF in the second exon and has been re-annotated as a protein-coding gene. Interestingly, the peptide was independently identified based on a proteomic strategy and termed SPAAR for small regulatory polypeptide of amino acid response.^23^ To validate the RNA-seq, *LINC00961* gene expression was evaluated by qRT-PCR in the same sample set used for RNA-seq, which demonstrated the same profile of expression (Supplementary material online, *Figure S2*). 

### 3.3 LINC00961/SPAAR gene silencing affects endothelial function

**Figure 3:**
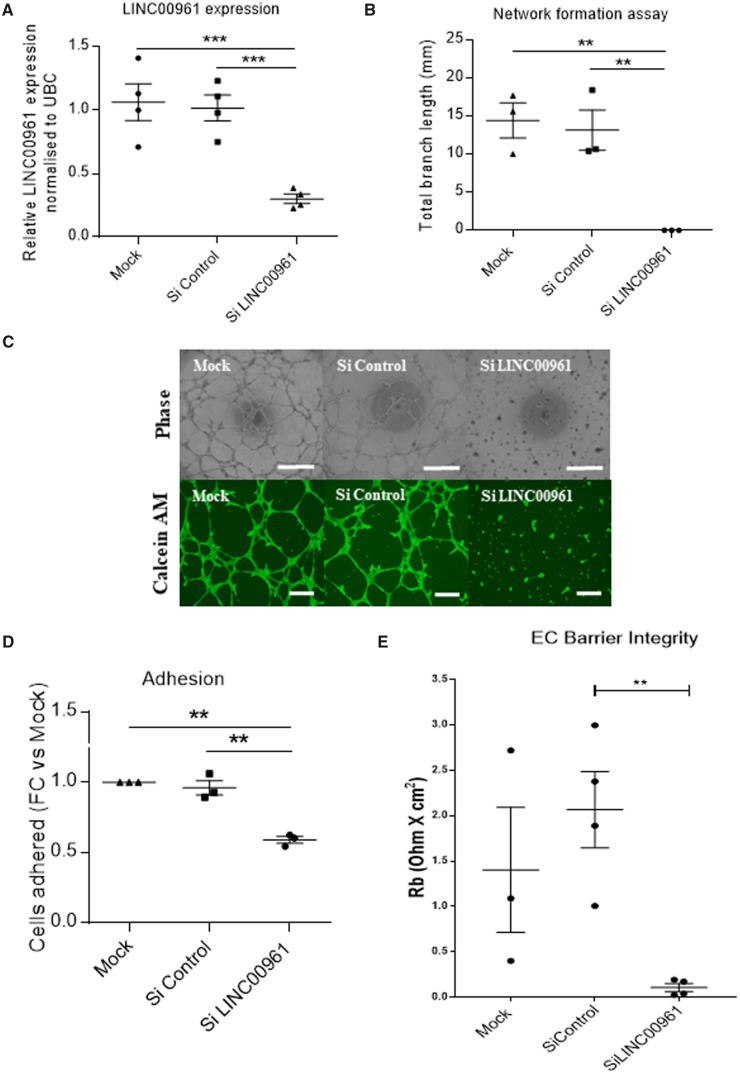
Functional impact of LINC00961/SPAAR depletion in ECs. (*A*) Confirmation of the dsiRNA-mediated depletion of *LINC00961* transcript in HUVECs by qRT-PCR (*n* = 4, unpaired *t*-test). (*B*) Network formation assay in *LINC00961* depleted HUVECs. Branch length assessed by Image J Angiogenesis plugin (*n* = 3, unpaired *t*-test). (*C*) Representative phase contrast and Calcein AM staining of network formation assay of *LINC00961* depleted and control HUVECs. Phase Scale bar = 0.5 mm. Calcein AM Scale bar = 0.1 mm. (*D*) Impact of *LINC00961* depletion on HUVEC adhesion (*n* = 3). (*E*) Analysis of average barrier resistance, expressed as Rb [Ohm × cm^2^], across a 10 h time course (*n* = 4 except for mock *n* = 3, one-way ANOVA). For data represented as fold change, the statistical analysis was done on the Log2 fold change using an one sample *t*-test. On the graphs, *P < 0.05 **P < 0.01 ***P < 0.001.

To assess the impact of silencing *LINC00961* transcript on endothelial function, we depleted *LINC00961* levels in HUVECs by 70%, utilizing dsiRNAs (*Figure 3A*). In an *in vitro* 2D Matrigel tubule network formation assay, *LINC00961* silencing resulted in attenuated branch formation (*Figure 3B, C*). Calcein AM was used to confirm that the lack of branch formation following *LINC00961* depletion was not a consequence of apoptosis (*Figure 3C*). We confirmed that *LINC00961* silencing did not affect cell viability using Annexin V and PI staining (Supplementary material online, *Figure S3A*). We then replicated the network formation phenotype via a GapmeR depletion strategy (Supplementary material online, *Figure S4*). Moreover, silencing *LINC00961* led to a significant reduction in cell adhesion (*Figure 3D*) and endothelial membrane barrier integrity (*Figure 3E* and Supplementary material online, *Figure S3B*). We also observed a trend towards a reduction in cell proliferation (Supplementary material online, *Figure S3C*) and migration (Supplementary material online, *Figure S3D*). To investigate whether *LINC00961* played a *cis*-regulatory role in the expression of the closely located gene HRCT1, we tested *HRCT1* transcript levels in siRNA *LINC00961* depleted cells. qRT-PCR analysis showed that *HRCT1* expression was unaltered by *LINC00961* modulation (Supplementary material online, *Figure S5A, B*). Similarly, siRNA silencing of *HRCT1* did not affect *LINC00961* levels (Supplementary material online, *Figure S5C, D*). 

### 3.4 Murine LINC00961/SPAAR locus knock out reduces adductor muscle capillary density following HLI

**Figure 4:**
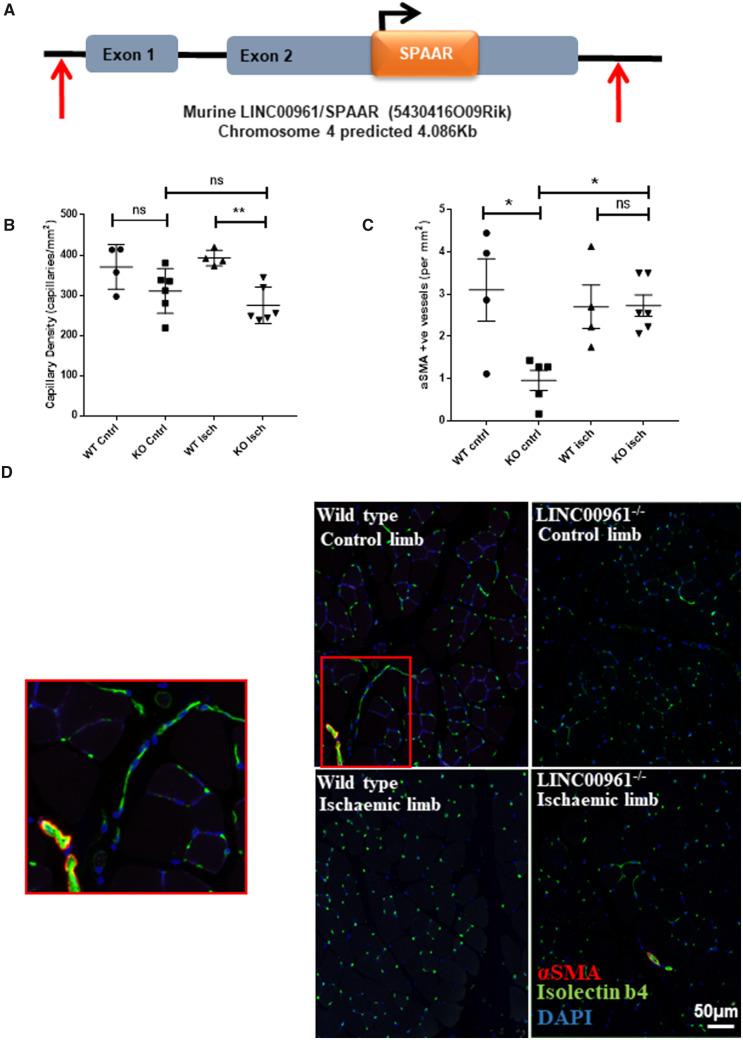
LINC00961/SPAAR KO mice have a reduced adductor muscle capillary density following HLI at 7 days. (*A*) Schematic representation of the deleted region of the LINC00961 mouse locus using CRISPR/Cas9 technology by Taconic©. Red arrows indicate the position of the guide RNA strands utilized to delete the whole locus. (*B*) Capillary density per sample. Five random regions of interest from three sections per sample were counted (*n* = 4 WT mice/6 KO mice, one-way ANOVA, ** *P* < 0.01, ns, not significant). (*C*) αSMA positive vessel density per sample. (*D*) Representative adductor muscle immunofluorescent images: Isolectin b4 (IB4) capillary/endothelium, αSMA, and nuclear DAPI, scale bar 50 µm. Zoomed panel on left corresponds to red box on area of WT control limb image.

To assess the role of the LINC00961 locus *in vivo*, we established a knockout (KO) mouse where the entire locus was deleted (*Figure 4A*). We first confirmed the absence of the LINC00961 mouse transcript by qRT-PCR (Supplementary material online, *Figure S6A*). We then tested the efficacy of injury-induced angiogenesis compared with wild type (WT) littermate controls at two time points. After 7 days, the capillary density between KO and WT animals was not significantly altered in the non-ischaemic leg (*P* = 0.2471). However, at 7 days after HLI LINC00961^−/−^ mice had a lower capillary density in the ischaemic adductor muscle compared with controls (*Figure 4B*). This was, therefore, comparable with the *in vitro* tubule formation data in *LINC00961* depleted HUVECs. Interestingly, KO animals had a significant decrease in the number of α-smooth muscle actin (αSMA) positive vessels at baseline compared with WT animals but this difference was not evident after injury (*Figure 4C*). We also analysed Laser Doppler ratio, capillary density, and αSMA positive vessels at 21 days. No significant differences at this later time point were observed (Supplementary material online, *Figure S6*). 

### 3.5 The LINC00961 locus encodes a biologically functional RNA

**Figure 5:**
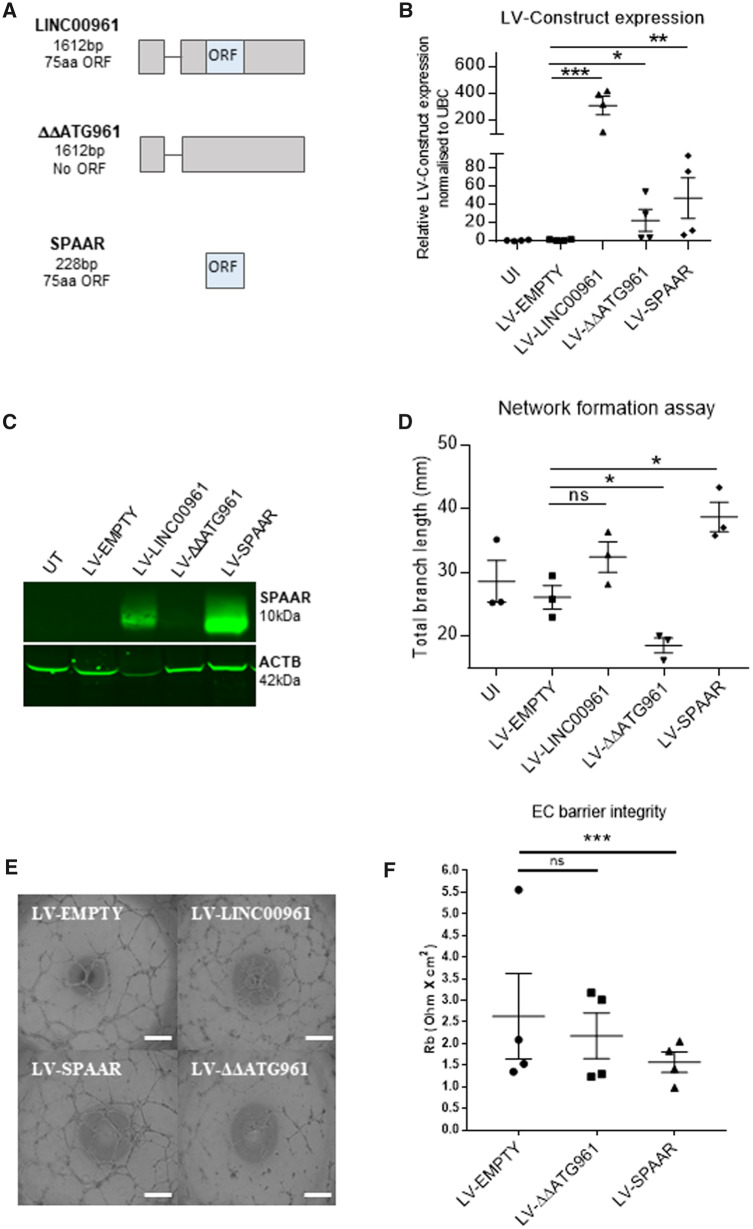
Impact of *LINC00961* transcript and SPAAR micropeptide overexpression in *in vitro* angiogenic assays. (*A*) Schematic representation of *LINC00961* LV constructs with transcript length in base pairs (bp) and encoded peptide length in amino acids (aa). (*B*) qRT-PCR validation of the LV constructs overexpression in HUVECs using primers targeting the ORF sequence. Unpaired *t*-test, comparison test vs. LV-EMPTY (*n* = 4). (*C*) Representative western blot of SPAAR micropeptide and β-actin in HUVECs infected with the LV constructs. (*D*) Network formation assay comparing HUVECs transfected with LV constructs. Branch length assessed by Image J Angiogenesis plugin. Unpaired *t*-test vs. LV-EMPTY (*n* = 3). (*E*) Representative Phase contrast of network formation assay of HUVECs transfected with LV constructs. (*F*) Analysis of average barrier resistance, expressed as Rb [Ohm × cm^2^], across a 10 h time course (*n* = 4, one-way ANOVA). Scale bar =0.5 mm. On the graphs, *P < 0.05, **P < 0.01, ***P < 0.001.

We next investigated the angiogenic effect of overexpressing either the full-length *LINC00961* transcript or the SPAAR ORF sequence in HUVECs, using lentiviral vectors (LV) (*Figure 5A*). We also generated a LV-ΔΔATG961 construct (*Figure 5A*), corresponding to the full-length transcript with mutations in the ORF initiation codons to block translation. qRT-PCR (*Figure 5B*) and western blotting (*Figure 5C*) confirmed overexpression. Overexpression of the LV-SPAAR construct significantly enhanced endothelial network formation, whereas LV-ΔΔATG961 produced opposite results, significantly inhibiting angiogenesis (*Figure 5D, E*). These data showed that the production of SPAAR induces network formation, whereas the *LINC00961* RNA alone possesses an inhibitory effect, independent of SPAAR micropeptide production, thus unveiling a bona fide lncRNA function for the *LINC00961* RNA. Furthermore, we observed that LV-mediated overexpression of SPAAR, but not the LINC00961 transcript, reduced endothelial barrier integrity (*Figure 5F,* and Supplementary material online,* Figure S7*). As cellular localization of lncRNA transcripts is informative with regards to their associated mechanisms, we determined the subcellular localization of *LINC00961* using RNA-fluorescent *in situ* hybridization (FISH) (Supplementary material online, *Figure S8A, B*) and cell fractionation (Supplementary material online, *Figure S8C*) and showed the presence of *LINC00961* in both the nucleus and the cytoplasm. 

### 3.6 Identification of binding partners for *LINC00961* RNA and SPAAR micropeptide

**Figure 6:**
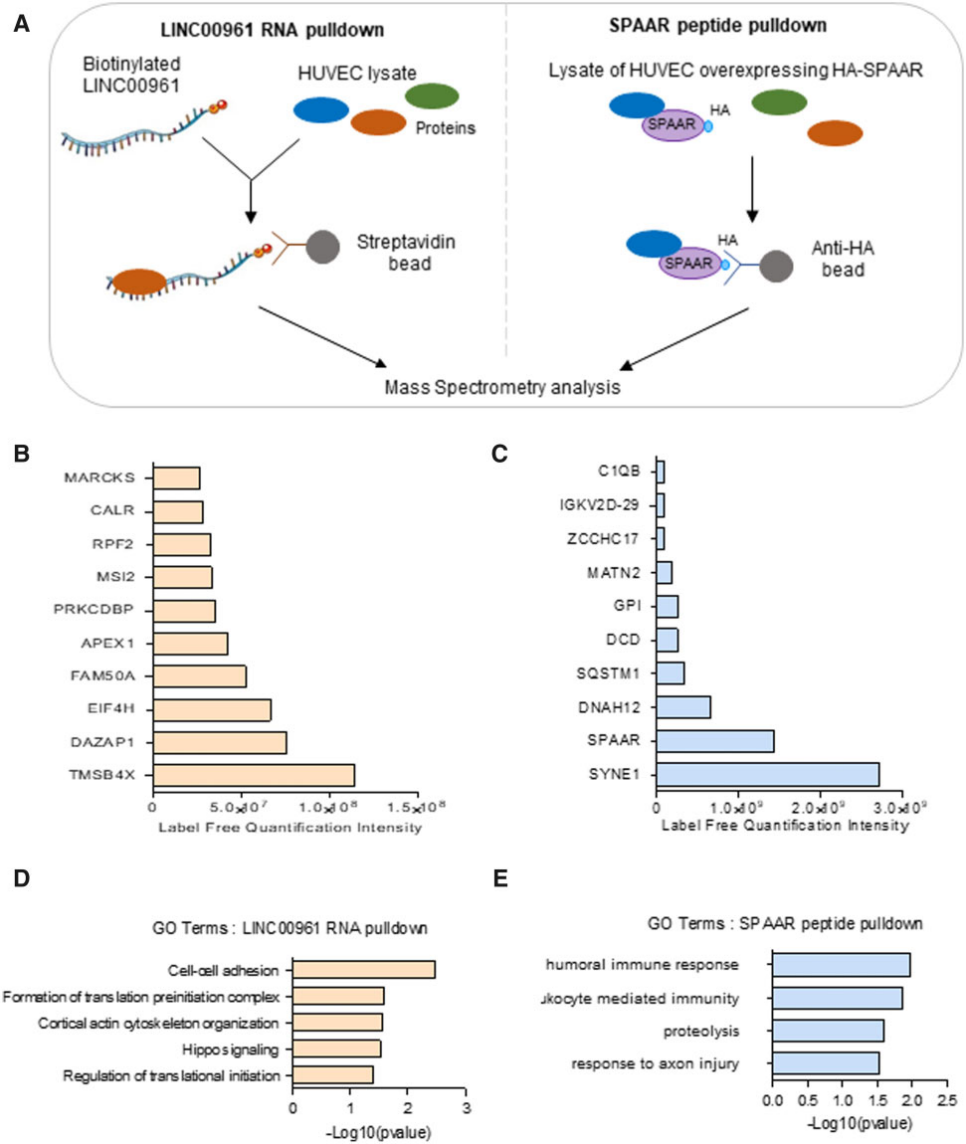
*LINC00961* and SPAAR both bind to actin-binding proteins. (*A*) Schematic of the *LINC00961* RNA and SPAAR peptide pull-down experiments in HUVECs. (*B*) List of the top 10 proteins identified in *LINC00961* RNA pull-down (ranked on label-free quantification value). (*C*) List of the top 10 proteins identified in HA-SPAAR peptide pull-down (ranked on label-free quantification value). (*D*) GO analysis on enriched proteins from *LINC00961* immunoprecipitation. (*E*) GO analysis on enriched proteins from *SPAAR* immunoprecipitation.

As both *LINC00961* and SPAAR are functionally relevant for ECs, we used RNA and protein pull-downs combined with mass spectrometry to identify the protein-binding partners of the lncRNA and SPAAR micropeptide in HUVECs (*Figure 6*). One hundred and forty-seven proteins were found in the *LINC00961* pull-down samples, which were not in the pull-down with the beads alone or the control GFP RNA (*Figure 6B* and Supplementary material online, *Tables S3* and *S4*). GO term analysis showed enrichment of terms related to cell–cell adhesion and cortical actin arrangement (*Figure 6D*). The top candidate was the G-actin sequestering molecule, thymosin beta 4-x (Tβ4) which is associated with reorganization of the actin cytoskeleton^28^ and is also involved in angiogenesis.^29^^,^^30^ Tβ4 functions within an actin organization pathway with other actin-associated molecules including Cofilin-1 and Profilin-1.^31^ Both Profilin-1 and Cofilin-1 were enriched in the *LINC00961* immunoprecipitation (Supplementary material online, *Table S3*); suggesting *LINC00961* may play a role in actin cytoskeleton remodelling. To confirm the interaction between *LINC00961* and Tβ4, we carried out immunoprecipitation of endogenous Tβ4 protein in HUVECs. qRT-PCR confirmed the detection of *LINC00961* in Tβ4 immunoprecipitation samples, thus independently validating an interaction of *LINC00961* with Tβ4 (Supplementary material online, *Figure S10A*). Immunofluorescence of Tβ4 in HUVECs confirmed the presence of Tβ4 in the cytoplasm in accordance with a plausible interaction with *LINC00961* (Supplementary material online, *Figure S10B*). 

We next identified protein-binding partners for SPAAR. We found 40 proteins enriched in the HA-SPAAR pull-down compared with the IgG pull-down controls and compared with the pull-downs in control cells not expressing the fusion protein (Supplementary material online, *Tables S5* and *S6*). GO analysis of SPAAR targets showed enrichment of terms related to immunity (*Figure 6E*). SPAAR has been previously shown to bind the v-ATPase complex in HEK293.^23^ However, these proteins were not found in the SPAAR pull-down in HUVECs, suggesting a different function for SPAAR in ECs. The top hit for SPAAR interactors was SYNE1, also known as NESPRIN-1, a regulator of EC shape and migration.^32^

### 3.7 Thymosin beta 4-x depletion phenocopies LV-ΔΔATG961 overexpression

**Figure 7:**
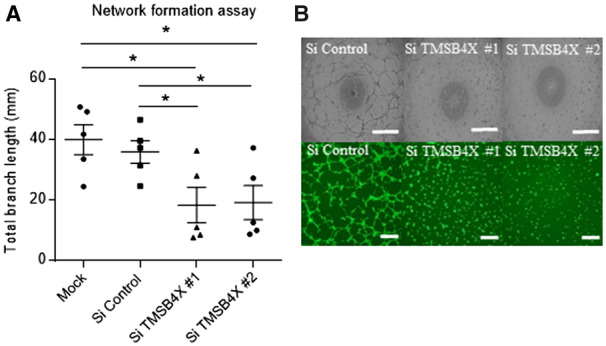
Thymosin beta 4-x KD in HUVECS has a similar phenotype to LV-ΔΔATG961 overexpression on tubule formation. (*A*) Network formation assay in dsiRNA-mediated *TMSB4X* depleted HUVECs. Branch length assessed by Image J Angiogenesis plugin, *n* = 5, unpaired *t*-test. (*B*) Representative phase contrast and Calcein AM staining of network formation assay of depleted HUVECs. Phase contrast Scale bar = 0.5 mm. Calcein AM Scale bar = 0.1 mm. On the graphs, *P < 0.05, **P < 0.01, ***P < 0.001.

To characterize the function of *LINC00961* and Tβ4 interaction, we assessed whether they co-regulated each other’s expression. siRNA silencing of *TMSB4X* (Supplementary material online, *Figure S9A*) did not alter *LINC00961* transcript levels (Supplementary material online, *Figure S9B*). Similarly, silencing *LINC00961* or overexpressing LV-ΔΔATG961 did not change *TMSB4X* transcript levels (Supplementary material online, *Figure S9C, D*). The known pro-angiogenic effect of Tβ4^29^^,^^30^ was confirmed in our system, with a 49% ± 16% reduction in network formation following *TMSB4X* depletion (*Figure 7A, B*). This reduction is similar to the overexpression of *LINC00961* transcript without the production of SPAAR micropeptide (LV-ΔΔATG961), suggesting that *LINC00961* lncRNA might negatively regulate Tβ4-mediated angiogenesis. 

## 4. Discussion

Using RNA-seq, we identified *LINC00961* as an endothelial enriched transcript. The strong impact on the endothelial phenotype following *LINC00961* level manipulations confirmed the relevance of our candidate selection using the combination of our hESC to EC RNA-seq with ENCODE RNA-seq datasets. This further highlights the need to investigate the role of lncRNA transcripts in endothelial biology.

In this study, we provide *in vitro* and *in vivo* evidence that the LINC00961 locus has a function in ECs. Whilst siRNA knock down (KD) *in vitro* affects many aspects of EC biology (angiogenesis, adhesion, proliferation, migration, and membrane integrity), we assessed the angiogenic role in a murine KO model. LINC00961^−/−^ mice had fewer αSMA positive vessels at day 7 baseline, suggesting a defect in the development, maturity, and or stability of larger vessels. After injury, KO mice have fewer capillaries at day 7, indicating a reduced capacity of the endothelium to undergo angiogenesis after injury. However, the effect of the KO was not observed by day 21 post-HLI. This suggests the KO animals may have a slower recovery rate after injury (due to an impairment in EC function), or activate compensatory mechanisms to maintain vessel numbers after injury. As we have a global KO, we cannot exclude the contribution of LINC00961 deletion in other cell types to this phenotype. To further investigate the role of this locus in EC behaviour, it would be worthwhile to switch to an EC-specific and conditional LINC00961 KO mouse model. In addition, it would be interesting to assess the effect of LINC00961 deletion in early development of vessel establishment and further characterize the dynamics of vessel recovery early in the HLI model.

Previous studies have outlined the role of the micropeptide SPAAR, encoded from the LINC00961 locus, during muscle regeneration.^23^ In our study, we showed opposing roles of *LINC00961* RNA and SPAAR micropeptide in angiogenesis, one being anti- and the other pro-angiogenic, respectively. The reduction in endothelial barrier integrity with SPAAR overexpression further validates our hypothesis that SPAAR is pro-angiogenic. Indeed, plastic junctions are required for sprouting angiogenesis.^33^ Therefore, It would be interesting to test the permeability of new SPAAR-induced vessels in an animal system using a plasma tracer.

To our knowledge, this is the first reported bi-functional locus in a cardiovascular setting. In other biological contexts, loci producing protein or functional ncRNAs through alternative splicing have been described.^34–36^ The novelty of the *LINC00961* locus is that the SPAAR micropeptide is produced from the functional *LINC00961* RNA instead of an alternative splicing transcript without an ORF. This configuration implies the requirement of a regulatory mechanism to control the levels of *LINC00961* RNA and SPAAR micropeptide independently of each other. The switch between *LINC00961* and SPAAR could be controlled at the translation level, similarly to the STORM micropeptide whose translation initiation is regulated by eIF4E phosphorylation.^37^ However, the functional activity of the lncRNA encoding the STORM micropeptide has never been demonstrated. Expression of the *LINC00961* transcript is high in basal HUVECs and detectable by qRT-PCR, in contrast, we are only able to see the presence of SPAAR micropeptide in LV-SPAAR conditions. This limitation is likely due to either very low protein levels in basal HUVECs or the detection limit of the antibody. The precise molecular control of *LINC00961* transcript and SPAAR levels needs further dissection in light of these findings.

We show that *LINC00961* RNA binds Tβ4, a well-established actin-binding protein with many additional functions including anti-inflammatory and anti-apoptotic properties, and a role in cell migration and angiogenesis.^28^ As *TMSB4X* transcript levels were not affected by *LINC00961* depletion, we propose that *LINC00961* regulates Tβ4 protein function. The enrichment of Profilin-1 and Cofilin-1, actin monomer-binding proteins, in the *LINC00961* immunoprecipitation suggests a potential role for *LINC00961* in actin recycling. Like Tβ4, Profilin-1 sequesters G-actin maintaining a large pool of monomeric actin. Unlike Tβ4 however, the high affinity of Profilin-1 for ATP allows it to act as a catalyst for the conversion of G-actin.ADP to G-actin.ATP, hence aiding the polymerization of G-actin to F-actin filaments.^38^ In fact, Profilin-1, Tβ4, and actin have been shown to produce a complex.^39^ This, alongside the fact that Cofilin-1 and Tβ4 have been shown to co-localize in multiple cells types, further validates the nature of their finely balanced roles in cytoskeletal dynamics.^40^^,^^41^ It would be of interest to dissect the interactions of these three proteins with *LINC00961* in future.

We show that SPAAR binds to SYNE1, another actin-binding protein, which suggests that the pro-angiogenic effects of SPAAR could be mediated through SYNE1 and the actin cytoskeleton. This is in contrast to our proposed mechanism of action of *LINC00961*, which may negatively affect actin cytoskeleton rearrangement through interaction with Tβ4. SYNE1 is involved in the cellular organization of organelles via connecting them to the actin cytoskeleton. SYNE1 is especially important as a member of the linker of nucleoskeleton and cytoskeleton complex which tethers the nuclear lamina to the actin cytoskeleton during nuclear positioning and cell polarization.^42^ Interestingly, SYNE1 is highly expressed in skeletal and cardiac muscle cells as it is essential in maintaining the characteristic peripherally located nuclei.^43^ Matsumoto and colleagues (2017) describe rapid muscle regeneration in mice lacking SPAAR; it would be interesting to ascertain if this phenomenon is in part mediated by an interaction, or lack thereof, between SPAAR and SYNE1. Furthermore, SYNE1 siRNA KD in HUVECs has been shown to reduce tubule formation in a Matrigel assay and decreased migration^34^^,^^44^ similar to our results with KD of the LINC00961/SPAAR locus.

Cytoskeletal remodelling is a dynamic process which is constantly being influenced by internal and external signals, with many actin-binding proteins having been identified.^45^ Here, we show that *LINC00961 and* SPAAR have independent actin-binding protein partners that could influence downstream cytoskeletal architecture. It will be of interest to investigate if, and how, lncRNA and micropeptide levels can change cellular behaviour through cytoskeletal changes.

In conclusion, our study provides important evidence for the expression and function of *LINC00961* in ECs. Our work shows a role for the *LINC00961* RNA, independent of the micropeptide SPAAR. This highlights the importance of a detailed bioinformatic and experimental approach to reveal the contribution of putative lncRNAs and their encoded proteins in cell behaviours.

## Authors’ contributions

A.H.B. conceived the study. A.H.B., H.L.S., R.S., M.B., and J.R. designed experiments and interpreted data. H.L.S., R.S, M.B., M.B., M.M., and C.R.P. performed experiments. J.R. performed the bioinformatics analysis, A.C., M.B., J.M., J.R., and A.H.B. supervised the research. A.H.B., H.L.S., R.S., and J.R. wrote the manuscript. All the authors discussed the data and edited the manuscript.

## Supplementary Material

cvaa008_supplementary_materials

## Notes

Translational perspective Treatment of ischaemic conditions remains a major cardiovascular health burden. Identification of genes and non-coding RNAs that regulate the function of the vascular endothelium is important to understand and evolve potential new strategies that might enhance vascular regeneration. Here, we describe and dissect the functional importance of a micropeptide-encoding RNA transcript in the vascular endothelium, and demonstrate that both the RNA and the peptide regulate endothelial biology. Modulation of this axis may be a novel approach to regulate angiogenesis.
