# Supplementary material for: The LINC00961 transcript and its encoded micropeptide, small regulatory polypeptide of amino acid response, regulate endothelial cell function
Source: Cardiovasc Res. 2020 Jan 28;116(12):1981–94. doi: 10.1093/cvr/cvaa008 (PMC8216332; doi:10.1093/cvr/cvaa008)
Supplement: cvaa008_supplementary_materials [file cvaa008_supplementary_materials.zip › cvaa008_Supplementary_Materials/LINC00961 Supplements dec 2019.pdf]

SUPLEMENTAL MATERIAL

DETAILED METHODS

Directed endothelial differentiation from human embryonic stem cells

A previously published protocol was employed to generate endothelial cells (ECs) from H9 human embryonic stem cells (hESCs) [1] . Briefly, hESCs, were dissociated into single cells using Tryple Select (Life Technologies, UK). 1×10^4 cells were seeded into a 5% Pluronic F-127 (Sigma-Aldrich, UK) coated 96- well round bottom microplates to generate embryoid bodies (EBs). The plates were centrifuged at 300 g for 3 minutes. Cells were cultured in Stemline II Hematopoietic expansion medium with the addition of 5 ng/mL Activin A (Peprotech, USA), 10 µmol/L Y-27632 (Millipore, Temecula, USA, 10 ng/mL vascular endothelial growth factor (VEGF) (Peprotech, USA), and 10 ng/mL bone morphogenetic protein 4 (BMP4) (R and D Systems, Minneapolis). After 2 days, the EBs were supplemented with Stemline II Hematopoietic expansion medium containing 10 ng/mL VEGF, 70 ng/mL Wnt3A, 140 ng/mL BMP4 and 35 ng/mL Activin A. On day 3, EBs were transferred to 0.1% gelatine-coated 6 well culture dishes (Sigma-Aldrich, UK) and resuspended in endothelial growth medium-2 (EGM-2) (Lonza, UK). The media was supplemented with 50 ng/ml of VEGF and the accompanying bullet kit, minus the VEGF and FBS supplement. Cells were maintained until day 7. Flow cytometry was performed as previously described [1]. Briefly, cell populations were dissociated by enzymatic digestion. Harvested cells were washed in PBS and incubated with specific antibodies (BD Bioscience, USA). FACS sorting was performed using anti-human CD326, anti human-CD56, anti human-CD31, and anti-human-CD144 by FACS Aria I or Aria III cell sorter (BD Biosciences, San Diego, USA. RNA was then extracted from these sorted populations.

Cell culture

Human saphenous vein endothelial cells (HSVECs) were obtained by enzymatic collagenase digestion of human saphenous veins (Ethics 15/ES/0094) and maintained in EC growth medium (EGM-2 BulletKit™) (Lonza, Basel, Switzerland) supplemented with foetal bovine serum (10%, Life Technologies, Paisley, UK) and Penicillin-Streptomycin (100U/ml) (Gibco, Paisley, UK). Human umbilical vein endothelial cells (HUVEC) (Lonza, Basel Switzerland) were cultured in the EC growth medium EGM2 (Lonza, Basel Switzerland) supplemented with 10% foetal bovine serum (FBS) (Life Technologies, Paisley UK) at 37ºC and 5% CO_2. All cells lines were used between passages 3 and 6, and kept at 37°C in a humidified atmosphere containing 5% CO_2.

dsiRNA and GapmeR transfections

GapmeRs were obtained from Exiqon (Denmark) and double stranded dicer-substrate short interfering RNA (dsiRNA) obtained from IDT (Leuven, Belgium) or Qiagen (UK). Sequences or product references are listed in Supplementary table 7. Transfection were performed using RNA iMAX (Life Technologies, USA).

RNA-Sequencing

RNA was obtained using the miRNeasy kit (Qiagen, U.K) from hESCs (4 replicates), the mesodermal and non-mesodermal population (4 replicates), the endothelial and non-endothelial population (2 replicates) and HSVECs (4 replicates). Ribosomal-depleted stranded libraries were prepared by Beckman Coulter Genomics and sequenced with an Illumina HiSeq at an average of 90 million reads per sample (paired end 2 x 100bp). Mapping was performed on the human genome reference sequence GRCh38, using Tophat version 2.0.10 [2] in conjunction with Bowtie version 1.0.0 based on a GRCh38 Ensembl transcriptome annotation. HTSeq was used to obtain a read count [3]. The differential expression was assessed using DESeq2 [4] and we considered a threshold of absolute Fold Change > 2 and adjusted p value < 0.01 to identify significant changes between two conditions. Sample clustering was evaluated using the Principal component analysis (PCA) tool available in DESeq2 on the regularized log

transformed data. The gene expression value, given as Fragments Per Kilobase of transcript per Million map read (FPKM) was obtained using Cufflinks [5]. Enriched genes in the day 7 endothelial population were obtained by selecting the significant changes versus the embryonic stem cell samples as well as the non-endothelial cell population. Genes with an expression above 2 FPKM were retained. The gene ontology analysis was carried out using topGO) on enriched genes over a background of expressed genes (FPKM>2 in at least one condition) (https://bioconductor.org/packages/release/bioc/html/topGO.html). Fisher's exact test was used to calculate p-values. GO terms were subsequently filtered to remove redundant terms using GO Trimming [6] with a soft threshold of 0.4. Heatmap of the z-score was generated thanks to the package 'pheatmap' (https://cran.r-project.org/web/packages/pheatmap/index.html). The z-score represents the deviation from the mean by standard deviation units of the Log2(FPKM+1) for each gene.

Transcript expression analysis

Total RNA was isolated using the miRNeasy kit (Qiagen, U.K). RNA was reverse transcribed using random primers, following manufacturer's instructions. Quantitative PCR (qPCR) was carried with TaqMan (Life Technologies, UK) or Power SYBR technologies (Life Technologies, UK). Ubiquitin C (UBC) was used as a reference gene. qPCR reactions were performed in technical duplicates with the QuantStudio 5 Real-time PCR system (Life Technologies, Paisley, UK). Relative quantification (RQ) was calculated using the 2-(ΔΔCT) method. The sequences of primers or TaqMan probes are provided in Supplementary Table 5.

Network formation assay

Network formation assay was performed using Matrigel (Corning, USA) according to the manufacturer's protocol. Briefly, 70 µl of Matrigel was used per well of a 96 well plate and allowed to set at 37 °C and 5 % CO_2 for 30 minutes. After which, 1 x 10^4 dissociated HUVECs were plated upon the Matrigel layer and incubated in EGM-2 (LONZA, UK) for 5.5 hours at 37 °C and 5 % CO_2. Calcein AM (Invitrogen) was used to determine cell viability. Tubule-like networks were visualised with a light microscope and phase images were taken at 4x magnification. Matrigel assays were performed in triplicate and total branch length was determined using angiogenesis analyser for Image J.

Proliferation assay

Proliferation was assessed using the Click-it EdU (5-Ethynyl-2´-deoxyuridine) 488 Proliferation assay (Life Technologies, UK) as per manufactures instructions. Briefly, HUVECs were seeded at a density of 1 x 10^5 per well of a 96 well plate 24 hrs prior to serum starvation conditions consisting of DMEM supplemented with 2% heat-inactivated FBS, 50 µg/mL penicillin, 50 µg/mL streptomycin, 2 mmol/L L-Glutamate and 1 mmol/L sodium pyruvate. After which, cells were induced to proliferate for 24 hrs by culturing in complete EGM-2 media in 10 % serum with 10 µM EdU. Cells were then dissociated and fixed in ice-cold 70 % ethanol for EdU flow cytometry analysis. EdU incorporation was confirmed using anti-EdU 488 antibody.

Migration assays

Migration assays were performed using an Electric Cell-substrate Impedance Sensing (ECIS) machine (Applied BioPhysics) as per Manufacturer's instructions. Briefly, 6 x 10^4 HUVECs were seeded into each well of an ECIS wound healing slide. The following conditions were used: electroporate 2500µA; wound: 30 second; frequency 40000. Migration ability was calculated after 24h by: velocity=r/t, where the radius is 125µm.

Cell viability assay

Cell viability assay (BioLegend) was performed using FITC Annexin V Detection Kit with PI (BioLegend) according to the manufacturer's protocol. In brief, cells were resuspended in 100 µl of binding buffer. 5 µl of Annexin V was added to the cell solution for 15 minutes before the addition of a further 400 µl of binding buffer. Prior to flow cytometry analysis 1:1000 of ToPro 3 (Life Technologies, UK), was added to the samples. Unstained and single stained samples were used to calibrate the setting on the flow cytometer. Samples were analysed on a BD LSR Fortessa Cell Analyzer (BD Biosciences, USA). The resulting data was analysed with FlowJo software (FlowJo LCC, USA).

Endothelial barrier integrity assay

An ECIS machine was used as per Manufacturer's instructions to measure resistance between cells in previously transfected cells. Briefly, 4 x 10^4 HUVECs were seeded into each well of an ECIS chamber side. Cells were allowed to adhere and then resistance was measured over 10 hours. Barrier resistance is expressed as Rb [Ohm x cm^2].

Lentiviral mediated production and induction

To generate lentiviral vectors, HEK293T cells were triple transfected with; a plasmid encoding the envelope of vesicular stomatitis virus (VSVg) (pMDG) (Plasmid Factory, Bielefeld, Germany), a packaging plasmid (pCMVΔ8.74) and pLNT/SFFV plasmid employing polyethylenimine (PEI; Sigma-Aldrich, USA) as previously described [1, 7]. Titres were determined by TaqMan qPCR. The following primers were used for the quantification: forward, 5'-TGTGTGCCCGTCTGTTGTGT-3'; reverse, 5'-GAGTCCTGCGTCGAGAGAGC-3'; probe, 5'- (FAM)- CAGTGGCGCCCGAACAGGGA- (TAMRA)-3. LINC00961 full-length, ΔΔATG961 and SPAAR sequences were generated by GeneART Gene synthesis (Life Technologies, UK). The sequences were cloned into pLNT/SFFV-MCS using the Kpn1 and XhoI sites (kind gift from Adrian J. Thrasher, London, UK).

Western blotting

HUVECs were washed with PBS and resuspended in 200 µl of RIPA buffer (Life Technologies, UK) supplement with cOmplete Mini, EDTA-free Protease Inhibitor Cocktail tablets (Roche, UK). Lysates were incubated on ice for 30 mins to allow complete lysis then were centrifuged at 15000 g for 10 mins at 4 °C to remove cell debris. Total protein concentration was determined using a Pierce™ BCA Protein Assay Kit (Thermo Fisher, UK) and a Perkin Elmer Victor 2 Microplate Reader. Western blots were conducted on the Life Technologies Bolt System as per manufacturer's instructions (Life Technologies, UK). 30 µg of protein with reducing Bolt loading buffer (Life Technologies, UK) was boiled for 5 minutes at 95°C. Samples were loaded into a 10 % bolt acrylamide gel (Life Technologies, UK). The proteins were transferred onto a nitrocellulose membrane and transferred at 10V for 1 hour. Membranes were blocked in 5% milk for 1 hour. SPAAR antibody (Cell Signalling) and β-Actin antibody (Abcam) were used at 1:1000 overnight for 4 °C. Secondary antibodies were used at 1:5000 (Li-Cor, UK). Detection was performed using the Li-Cor Odyssey Blot System (Li-Cor, UK). Protein loading levels were determined using the REVERT total protein stain normalisation protocol as per manufacturer's instructions (Li-Cor, UK).

RNA fractionation

RNA fractionation was performed as previously described [1] and as per manufacturer's instructions (Paris Kit, Life Technologies, UK). Briefly, HUVECs were dissociated and homogenized in ice-cold Fractionation Buffer and incubated for 5 minutes at 4˚ C. Nuclei were separated from the cytoplasmic fraction by centrifugation. Intact nuclei were then lysed using the Cell Disruption Buffer. The lysed cellular components were mixed in equal volumes with Lysis/Binding solution and applied to a filter containing cartridge. The RNA was then washed three times before eluting in nuclease free water. For cDNA synthesis, equal volumes of nuclear and cytoplasmic RNA were used. *NEAT1* RNA expression in the fractions was used to confirm effective nuclear-cytoplasmic separation.

RNA fluorescence in situ hybridization (FISH)

20 tiled digest oligo probes targeting both exons of LINC00961 were custom generated. RNA-FISH was performed following the manufacturer's instructions (QuantiGene ViewRNA cell ISH cell assay, Life Technologies, UK). For spatial localisation of LINC00961, SNORD3 and UBC were used as controls (Life Technologies, UK). 1 x 10^4 HUVECs were seeded on 0.2 % gelatin coated 16-mm coverslips until 80 % confluency. After which the cells were washed and fixed in 4 % formaldehyde supplemented with 1 % glacial acetic acid. Detergent QS was used to permeabilise the cells and following a 1:6000 protease digest, cells were incubated with a combination of LINC00961 probe and UBC or SNORD3. Unstained cells, incubated with only the probe set buffer served as a negative control. The presence of SNORD3 indicated the permeabilisation of the nucleus. After probe hybridisation, cells were incubated with pre-amplifier for 1 hr and, then amplifier for 30 minutes. Following incubation with fluorescent probes, samples were counterstained with DAPI mounting medium (Vectorshield, UK). Staining was visualised by Andor Revolution XDi spinning disk confocal microscope. Z-stacking confirmed staining within the nucleus. Confocal images were generated with the assistance of University of Edinburgh's Confocal and Advanced Light Microscopy Facility.

Biotinylated LINC00961 RNA pull-down

Biotinylated RNA was *in vitro* transcribed using the T7 RiboMAX Express Large Scale RNA Production System (Promega, UK) as per manufacturer's instructions. Briefly, 1 µg of the cDNA template was used as a template for T7 RNA polymerase *in vitro* transcription and incubated at 37 °C for 30 minutes. The primers used are detailed in Supplementary Table 7. The generated RNA was extracted using the miRNeasy kit (Qiagen, UK) following manufacturer's instructions. The resulting RNA had pCp–Desthiobiotin (Jena Biosciences, UK) attached to the 3'end of the RNA strand via T4 RNA ligase and Pierce RNA 3' End Desthiobiotinylation kit (ThermoScientific, UK). Biotinylated lncRNA was incubated with streptavidin magnetic beads and 20 µg of HUVECs protein lysate. This was performed following the manufacturer's instructions using the Pierce Mag RNA Protein Pulldown kit (Thermo Scientific). Non-specific interactions were removed by stringent washes and the leftover lncRNA-binding proteins were eluted off the beads. The elution buffer was non-denaturing. Samples were analysed by liquid chromatography mass spectrometry (LC-MS).

Mass spectrometry

Eluting peptides were ionised at +2kV before data-dependent analysis on a Thermo Q-Exactive Plus. MS1 was acquired with mz range 300-1650 and resolution 70,000, and top 12 ions were selected for fragmentation with normalised collision energy of 26, and an exclusion window of 30 seconds. MS2 were collected with resolution 17,500. The AGC targets for MS1 and MS2 were 3e6 and 5e4 respectively, and all spectra were acquired with 1 microscan and without lockmass. Finally, the data were analysed using MaxQuant (ver. 1.5.2.8) in conjunction with Uniprot Fasta database, with match between runs (MS/MS not required), LFQ with 1 peptide required, and statistical analyses performed in R. Subtractive proteomics was performed to identify proteins in the LINC00961 pull-downs compared to the GFP control and/or beads only pull-downs (n=2). Identified peptides were considered only if they have over 2 unique peptides and were chosen based on LFQ. These candidates were taken forward for targeted immunoprecipitation.

Thymosin beta 4-x immunoprecipitation

Tβ4 immunoprecipitation was performed as previously described [8]. Briefly, five 10cm^2 dishes of HUVECs were crosslinked with 0.4% formaldehyde for 10 minutes and then neutralised with 1 M glycine. Cells were harvested by scraping and lysing with Triton X-100-supplemented hypotonic buffer [10 mM Tris-HCL pH 7.5, 10 mM NaCl, 10 mM EDTA, 0.5% Triton X-100, and cOmplete Mini, EDTA-free Protease Inhibitor Cocktail tablets (Roche, UK)]. The supernatant was then centrifuged and NaCl adjusted to 150mM. 10 % of the total supernatant was removed for downstream RNA extraction. Supernatants were pre-cleared with 20 µl of

Dynalbeads G (Life Technologies, UK) at 4°C for 1 hr using an orbital rotator. Beads were then removed using a DynaMag and the supernatant was transferred to a fresh tube. 20 µl of Dynalbeads G and 4 µg Anti-TMSB4X antibody (Santa Cruz Biotechnology, UK) was introduced into the supernatant, which was further supplemented with RNase inhibitors. Samples were incubated at 4°C for 2 hrs on an orbital rotator. Using the DynalMag magnet, the bead bound complexes were washed 5 times with NET-2 buffer (NET-2 buffer [50 mM Trsi-HCl pH 7.4, 200 mM NaCl, 0.05 % NP40, and cOmplete Mini, EDTA-free Protease Inhibitor Cocktail tablets, RNase inhibitors). After the final wash was removed, the bound complexes were de-crosslinked by incubation at 70 °C for 1 hour before RNA extraction.

Immunofluorescent staining

Adductor muscle was paraffin embedded and 5µm sections were taken. Tissues were stained with isolectin-B4 to show capillaries (endothelium) (Thermo Fisher) and α-smooth muscle actin (α-SMA) (Sigma) to show smooth muscle and pericyte cells (arteries/arterioles). Tissue was imaged using either a Zeiss LSM 780 or Slide scanner Axio Scan.Z1, Zeiss. Five regions of interest ($0.42\ \mu m^2$) were taken from 3 sections of each adductor muscle. Capillary density and αSMA positive vessels were quantified utilising Image J software and created a macro to detect positive (capillaries) which we used on the green channel only of each image:

```
run("Subtract Background...", "rolling=50");
setOption("BlackBackground", false);
run("Make Binary");
run("Watershed");
run("Analyze Particles...", "size=0.03-Infinity show=Ellipses exclude include summarize in_situ");
```

Images were analysed with a size limit of particles set to 0.02 or 0.03.

Cells were fixed for 30 min at room temp with 4% paraformaldehyde and washed with phosphate buffered saline (PBS). Next, cells were permeabilised with 0.1% Triton-X-100 in PBS for 5 min at room temperature. A furtherPBS wash was carried out and cell were then blocked for 30 min at room temperature with 5% goat serum in PBS. Tβ4 antibody (Abcam 14335) was prepared in 5% goat serum (1/200) and cells incubated for 1 hour at 37°C. The primary antibody solution was washed off using PBS before cells were incubated with a secondary antibody (goat anti rabbit Alexa-Fluor-488, Abcam 150077) diluted 1/400 in 5% goat serum for 45 min at 37°C, protected from light. Finally, cells were washed with PBS and then mounted with ProLong Gold containing DAPI (Thermo Fisher). Images were taken on a Zeiss LSM 780 confocal microscope.

SUPPLEMENTAL REFERENCES

1. Boulberdaa M, Scott E, Ballantyne M, Garcia R, Descamps B, Angelini GD, Brittan M, Hunter A, McBride M, McClure J, Miano JM, Emanueli C, Mills NL, Mountford JC, Baker AH. A Role for the Long Noncoding RNA SENCR in Commitment and Function of Endothelial Cells. *Mol Ther* 2016;**24**:978-990.
2. Kim D, Pertea G, Trapnell C, Pimentel H, Kelley R, Salzberg SL. TopHat2: accurate alignment of transcriptomes in the presence of insertions, deletions and gene fusions. *Genome Biol* 2013;**14**:R36.
3. Anders S, Pyl PT, Huber W. HTSeq--a Python framework to work with high-throughput sequencing data. *Bioinformatics* 2015;**31**:166-169.
4. Love MI, Huber W, Anders S. Moderated estimation of fold change and dispersion for RNA-seq data with DESeq2. *Genome Biol* 2014;**15**:550.
5. Trapnell C, Roberts A, Goff L, Pertea G, Kim D, Kelley DR, Pimentel H, Salzberg SL, Rinn JL, Pachter L. Differential gene and transcript expression analysis of RNA-seq experiments with TopHat and Cufflinks. *Nat Protoc* 2012;**7**:562-578.
6. Jantzen SG, Sutherland BJ, Minkley DR, Koop BF. GO Trimming: Systematically reducing redundancy in large Gene Ontology datasets. *BMC Res Notes* 2011;**4**:267.
7. Ballantyne MD, Pinel K, Dakin R, Vesey AT, Diver L, Mackenzie R, Garcia R, Welsh P, Sattar N, Hamilton G, Joshi N, Dweck MR, Miano JM, McBride MW, Newby DE, McDonald RA, Baker AH. Smooth Muscle Enriched Long Noncoding RNA (SMILR) Regulates Cell Proliferation. *Circulation* 2016;**133**:2050-2065.
8. Elbarbary RA, Li W, Tian B, Maquat LE. STAU1 binding 3' UTR IRAlus complements nuclear retention to protect cells from PKR-mediated translational shutdown. *Genes Dev* 2013;**27**:1495-1510.

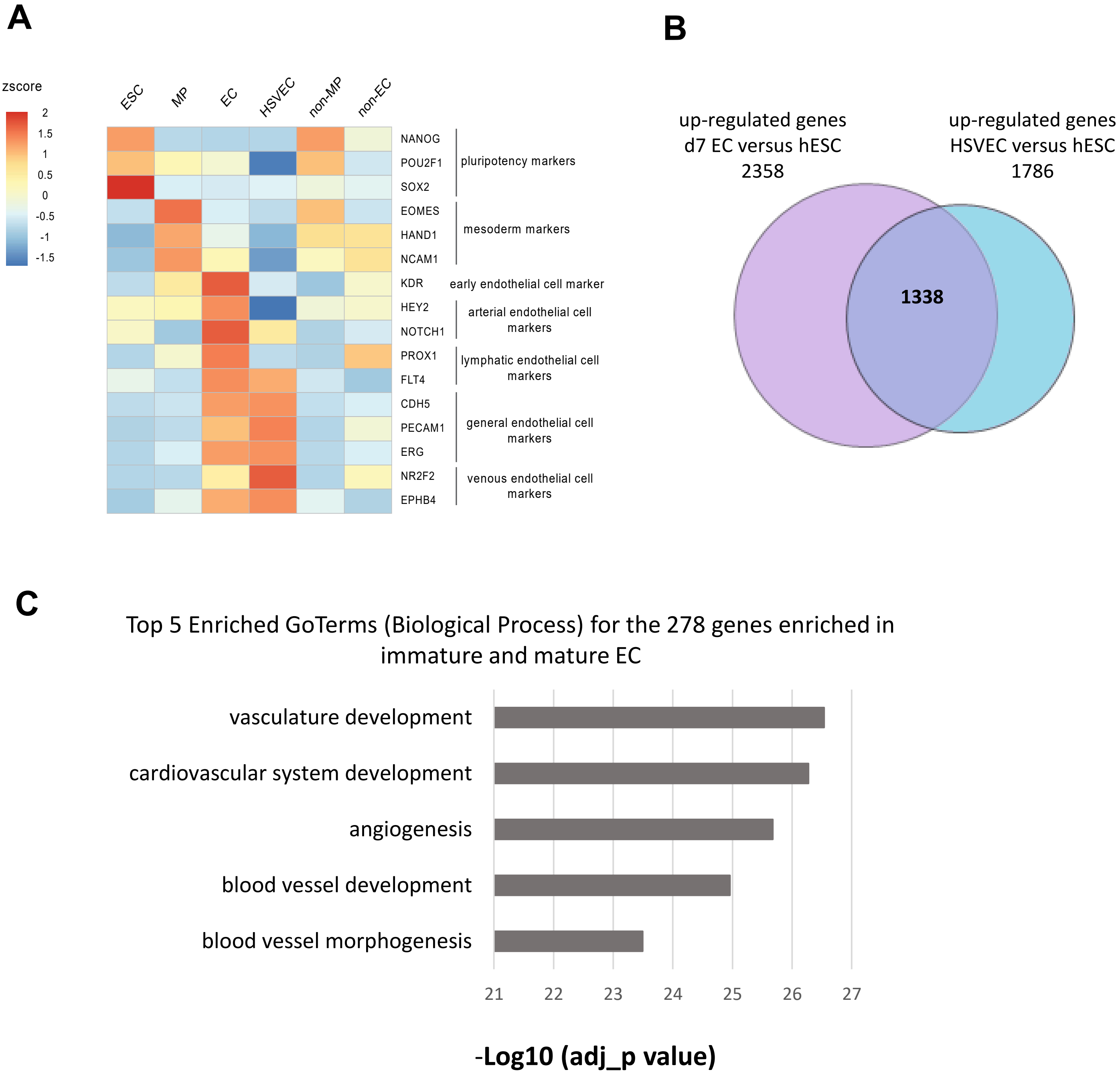

Supplementary Figure 1: Validation and characterization of the endothelial cell identity of the day7 EC population.(A) Heatmap (as z-score of Log2(FPKM+1)) showing the down-regulation of pluripotency associated genes and the up-regulation of mesoderm and endothelial specific transcripts. The rows are displayed based on a hierarchical clustering. The different conditions are day 0 H9 hESC (ESC); Day 3 mesodermal population CD326^{low}CD56^{+} (MP); Day 3 remaining population (non-MP); Day 7 EC CD144^{+}CD31^{+}(EC); Day 7 Remaining population (non-EC); Human Saphenous vein endothelial cell (HSVEC). (B) Venn diagram showing the overlap of up-regulated genes between d7 EC *versus* hESCs, and HSVECs *versus* hESCs. (C) GO analysis on the genes enriched in the d7 EC population. The graph shows the top 10 biological GO terms ranked by p-value.

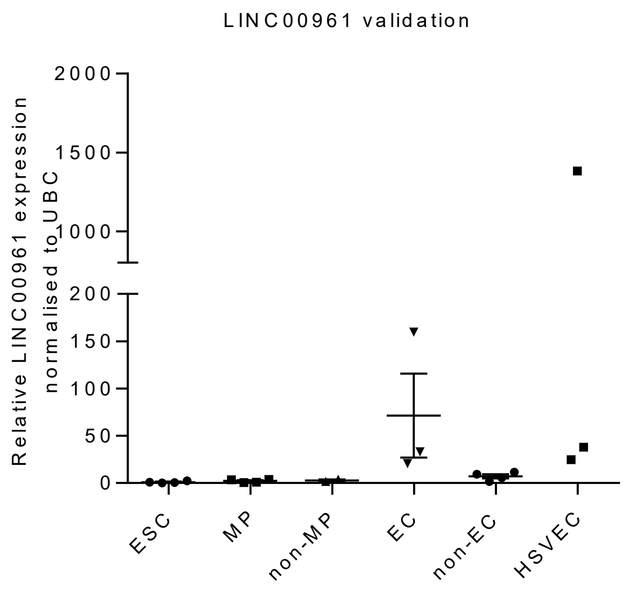

Supplementary Figure 2: Validation of LINC00961 expression in day7 population. qRT-PCR showed that day7 EC and HSVEC positive control samples were both enriched for *LINC00961* transcript expression.

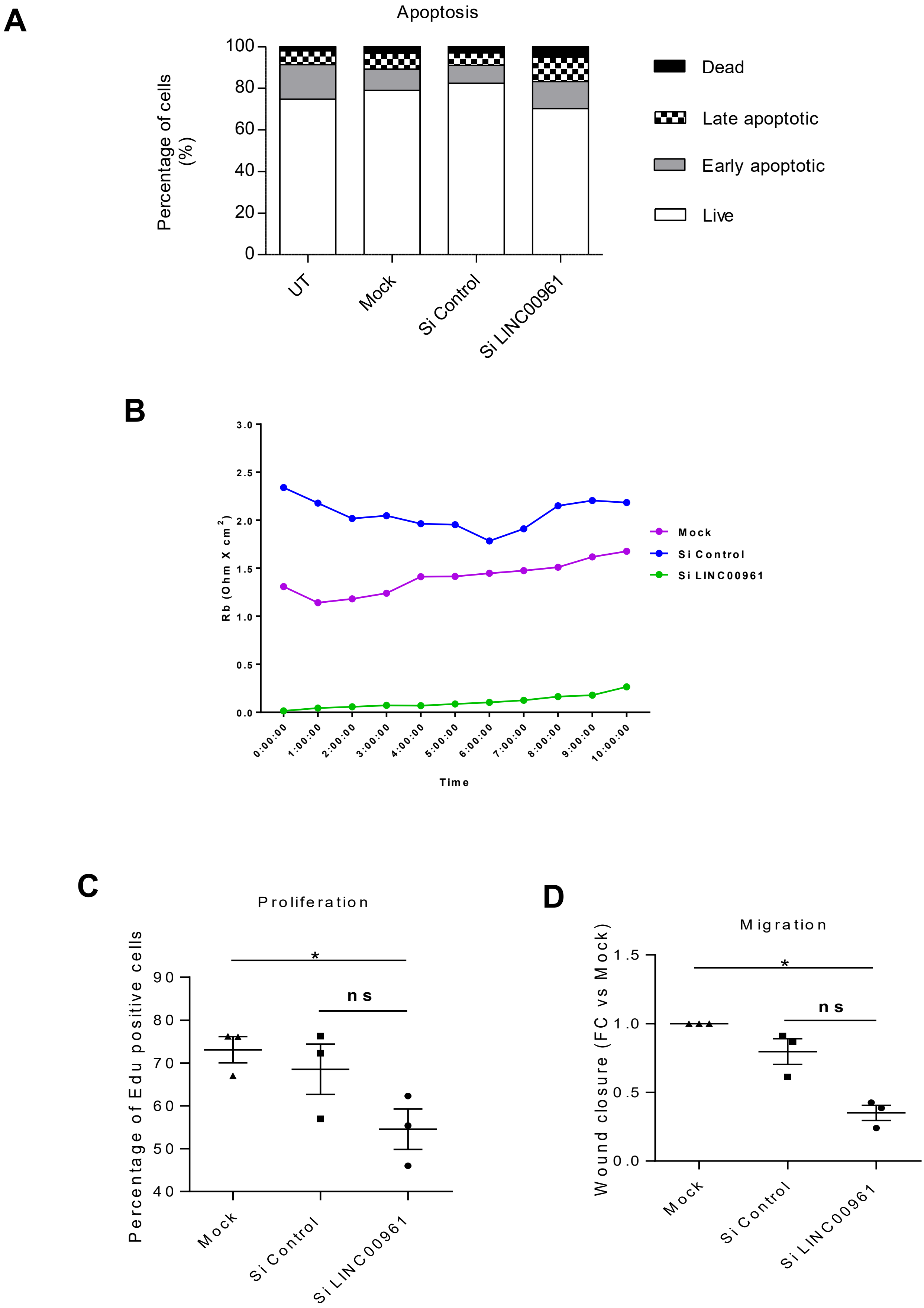

Supplementary Figure 3: Effect of LINC00961/SPAAR depletion on apoptosis and endothelial membrane integrity. (A) Impact of dsiRNA-mediated LINC00961/SPAAR depletion on cell death as assessed by Annexin V and PI staining compared to controls. (B) Trace of average barrier resistance between cells, in ohm x cm^2 (Rb), over a 10 hour period, n=4 except for mock n=3. (C) Impact of *LINC00961* depletion on proliferation based on EdU incorporation, n=3. (D) Impact of *LINC00961* depletion on migration, n=3. On the graphs, * $p<0.05$, unpaired t-tests.

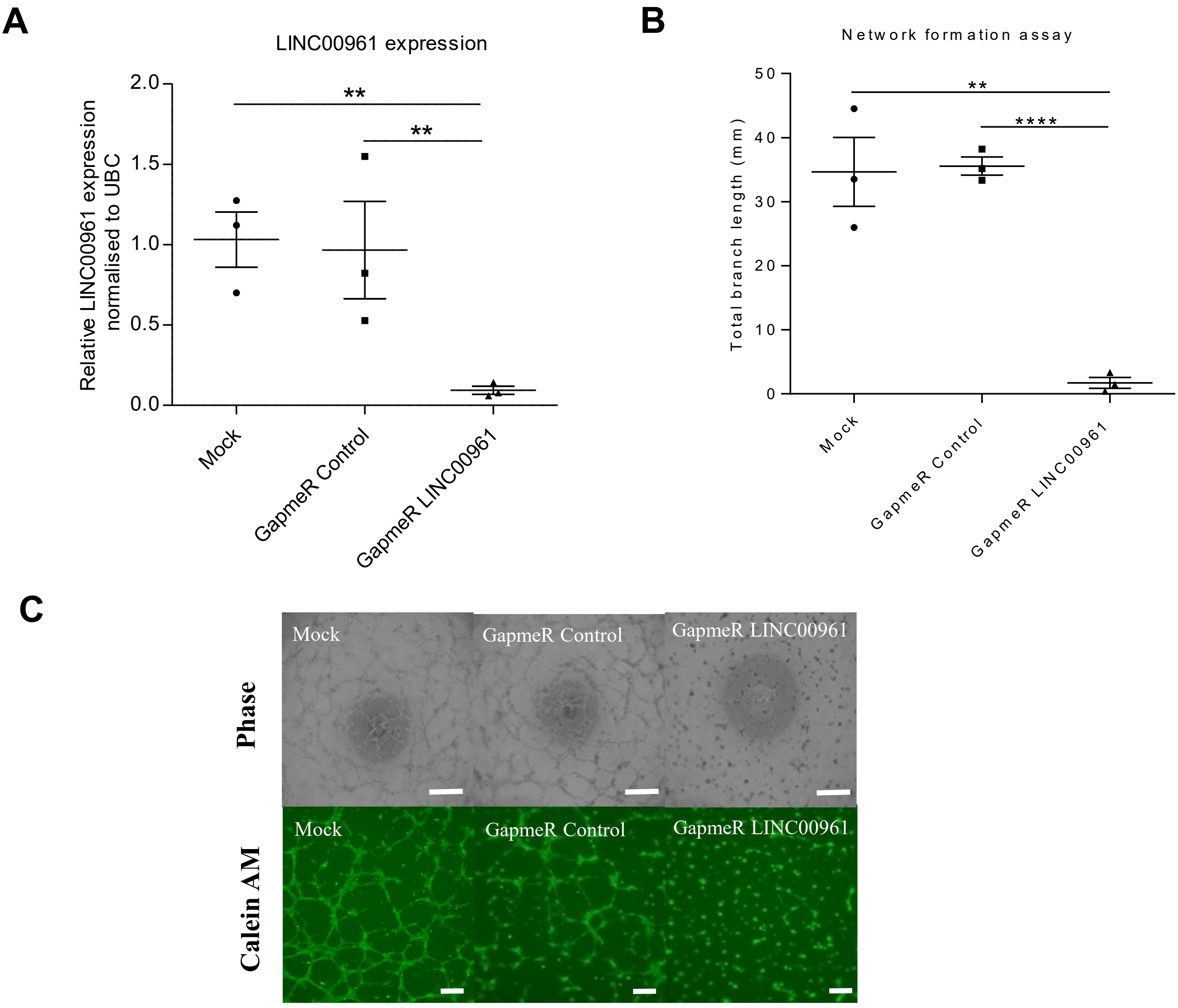

Supplementary Figure 4: Confirmation of the network formation phenotype of LINC00961/SPAAR knock down using GapmeR mediated depletion. (A) qRT-PCR of *LINC00961* in GapmeR depleted HUVECs (compared to GapmeR control and mock transfected cells), n=3.(B) Network formation assay in *LINC00961* depleted HUVECs. Branch length assessed by Image J Angiogenesis plugin., n=3. (C) Representative phase contrast and Calcein AM staining of network formation assay of *LINC00961* depleted HUVECs compared to controls. Phase Scale bar =0.5mm. Calcein AM Scale bar =0.1mm. On the graphs, **p<0.01 **** p<0.0001, unpaired t-tests.

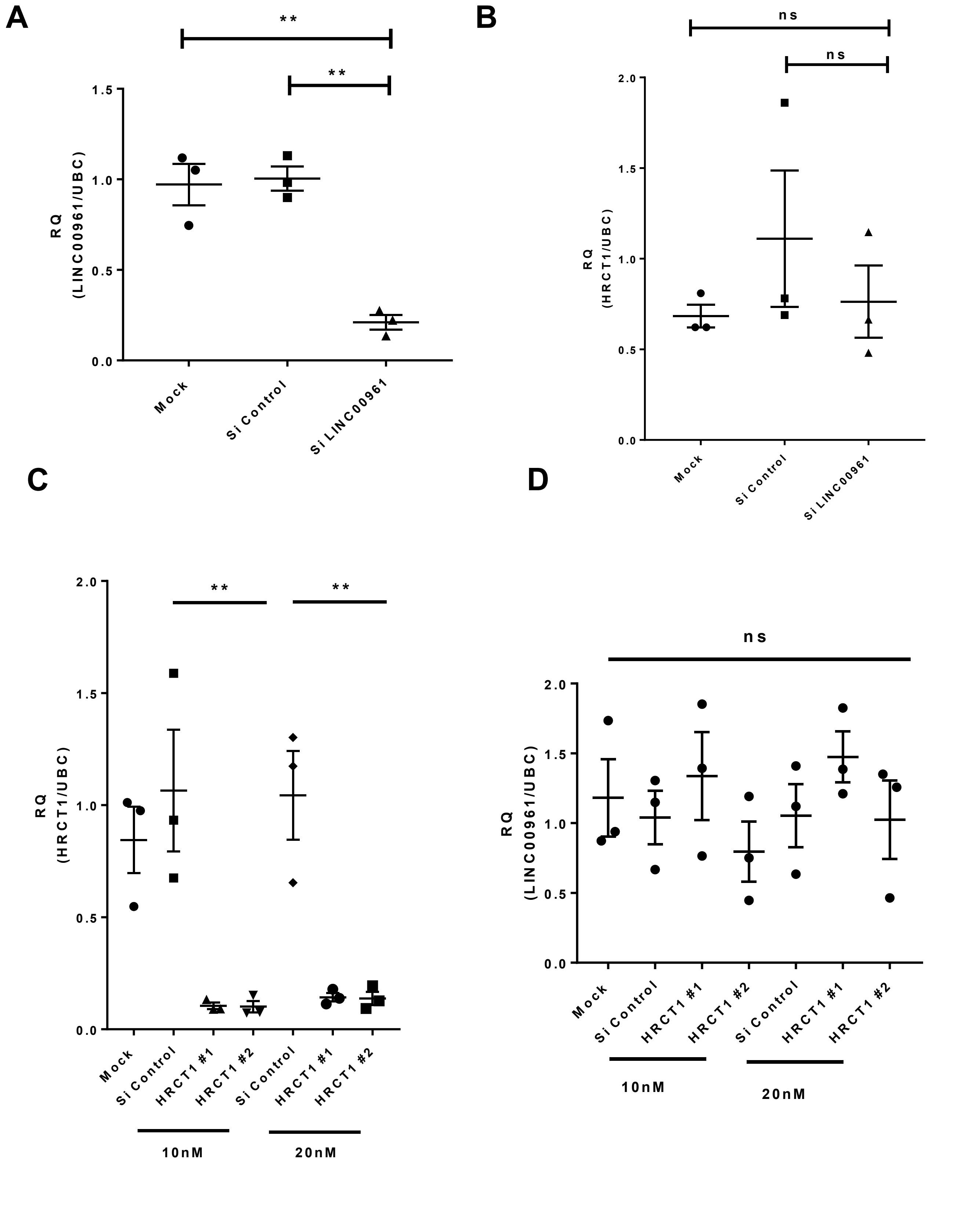

Supplementary Figure 5: *LINC00961* transcript does not regulate *HRCT1* transcript level and *vice versa*. (A) Confirmation of *LINC00961* KD by qRT-PCR on dsiRNA treated HUVECs (compared to dsiRNA control and mock transfected cells), n=3. (B) Impact of *LINC00961* dsiRNA on *HRCT1* transcript expression as assessed by qRT-PCR n=3. (C) Confirmation of *HRCT1* KD by qRT-PCR, n=3. (D) Impact of *HRCT1* dsiRNA on *LINC00961* transcript expression as assessed by qRT-PCR, n=4. On the graphs, ns = not significant. **p<0.001, unpaired t-tests.

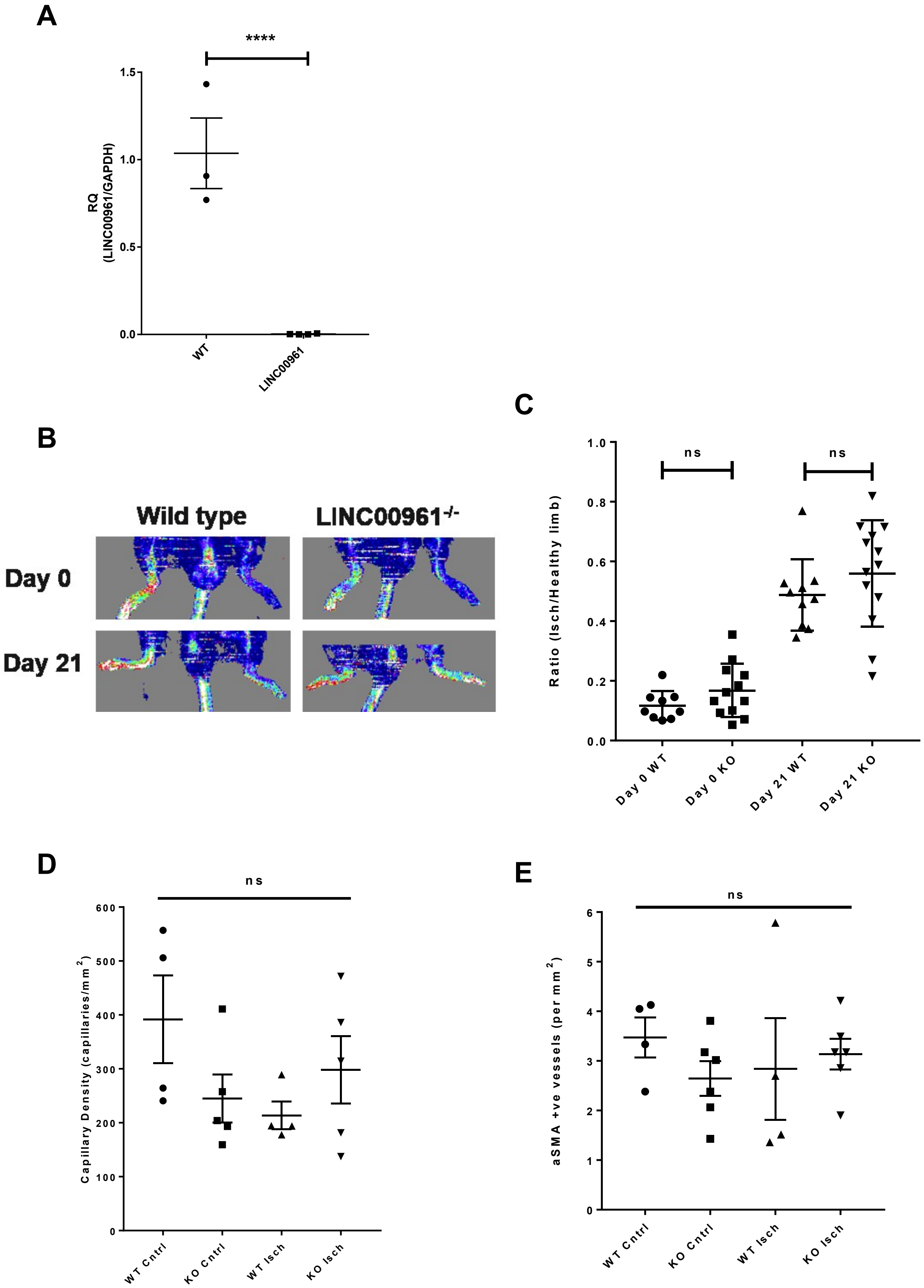
A
****
RQ
(LINC00961/GAPDH)
1.5
1.0
0.5
0.0
WT
LINC00961
B
Wild type
LINC00961-/-
Day 0
Day 21
C
ns
ns
Ratio (Isch/Healthy limb)
1.0
0.8
0.6
0.4
0.2
0.0
Day 0 WT
Day 0 KO
Day 21 WT
Day 21 KO
D
ns
Capillary Density (capillaries/mm2)
600
500
400
300
200
100
0
WT Cntrl
KO Cntrl
WT Isch
KO Isch
E
ns
aSMA +ve vessels (per mm2)
6
5
4
3
2
1
0
WT Cntrl
KO Cntrl
WT Isch
KO Isch

F

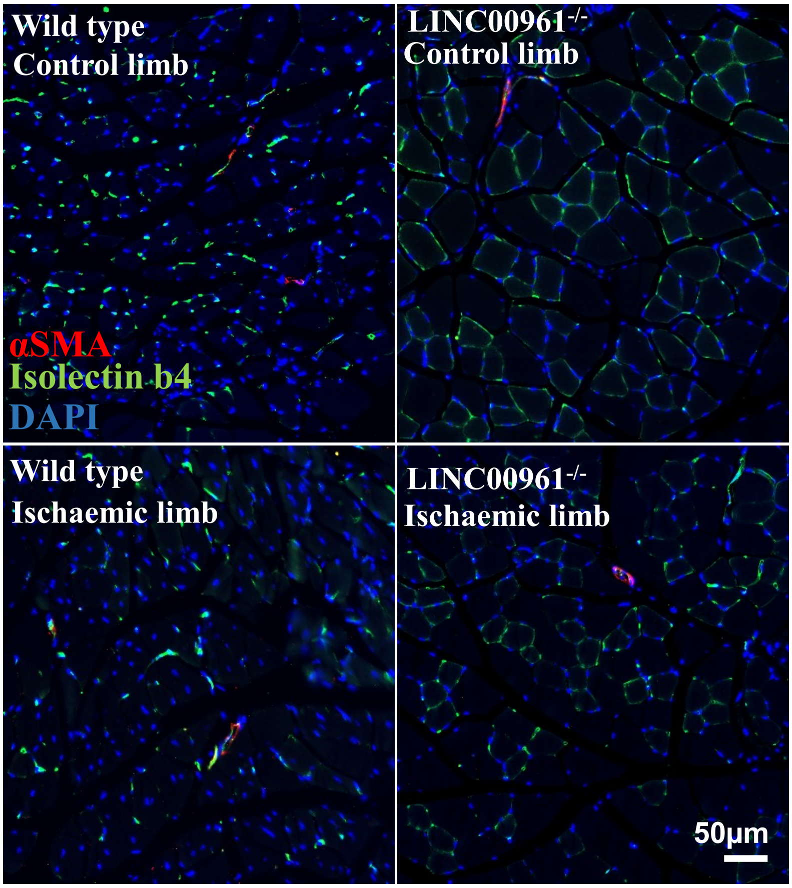

Supplementary Figure 6: *LINC00961$^{-/-}$* animals show no differences in vessel densities after 21 days hind limb ischemia. (A) Confirmation of *LINC00961* deletion in knock out animals. *LINC00961* transcript expression as assessed by qRT-PCR in whole kidney tissue, n= 3 WT/ 4 KO **** p<0.0001, unpaired t-test. (B) Representative Laser Doppler images of WT and KO animals immediately after ischemia induction and after 21 days. (C) Graph shows the ratio of blood flow to the ischemic paw compared to the control paw of WT and KO animals after surgery and after 21 days (D) Capillary density per sample. Five random regions of interest from 3 sections per sample were counted (n= 4 WT /5 KO, one-way ANOVA, ns = not significant). (E) αSMA positive vessel density per sample (F) Representative adductor muscle immunofluorescent images: Isolectin b4 (IB4) capillary/endothelium, αSMA, and nuclear DAPI, scale bar 50µm.

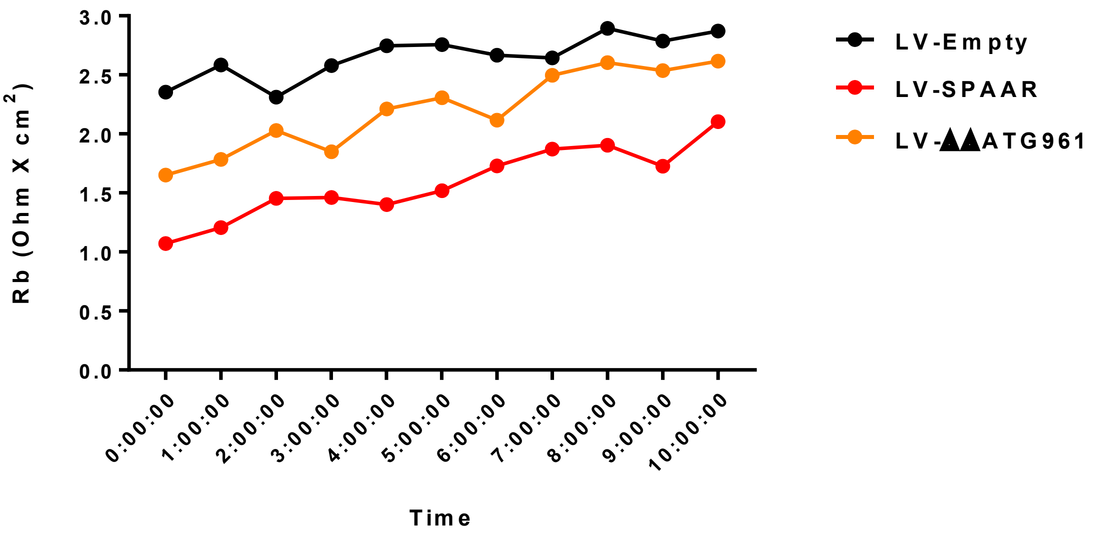

Supplementary Figure 7: : SPAAR micropeptide overexpression but not LINC00961 transcript expression affects endothelial barrier integrity. Impact of *LINC00961* (without SPAAR) and SPAAR only lentiviral construct overexpression in HUVEC endothelial barrier integrity over a 10 hour period, n= 4.

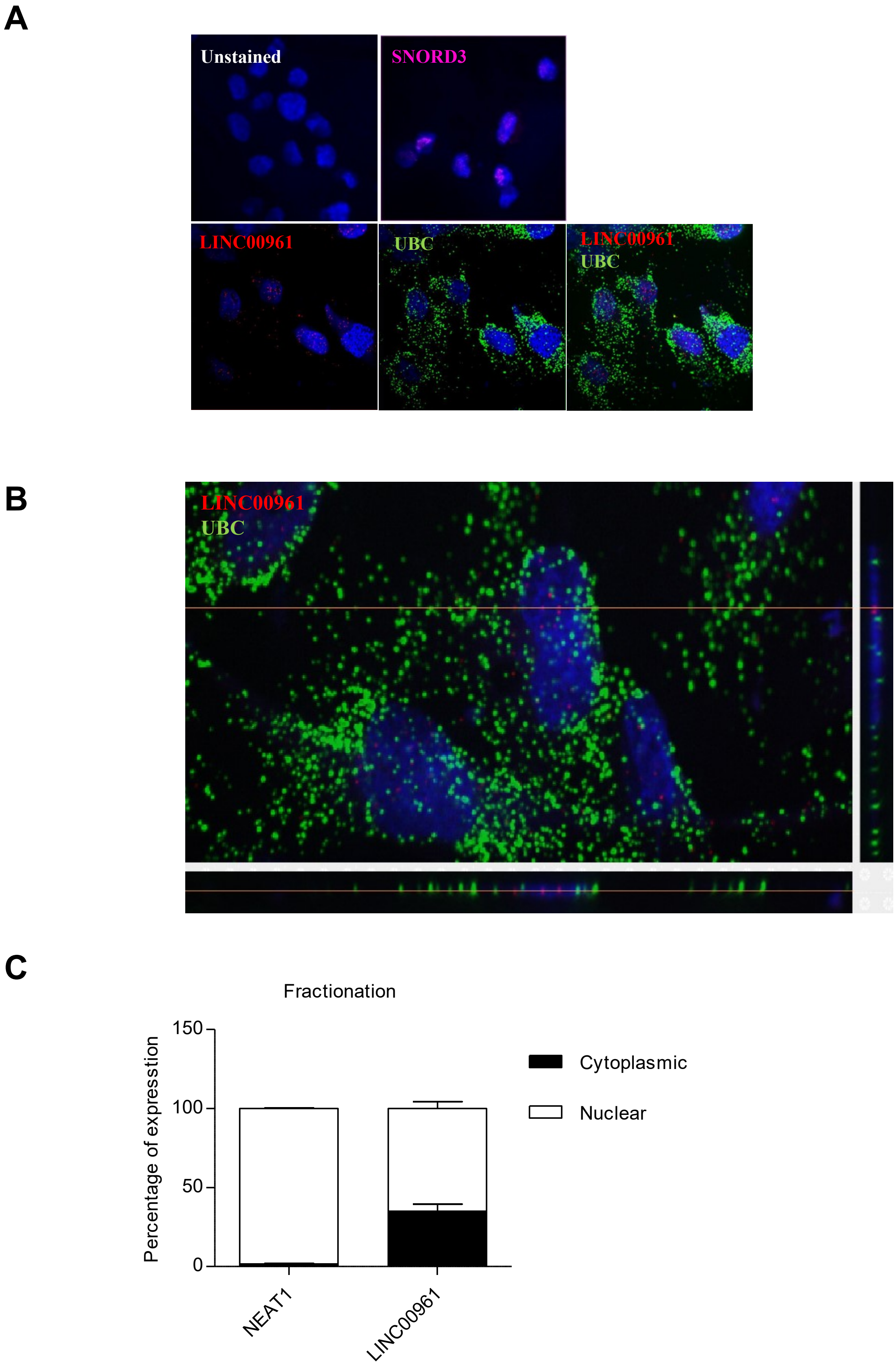

Supplementary Figure 8: Endogenous localisation of the *LINC00961* transcript. (A) RNA-FISH of *LINC00961* in HUVECS. *UBC* mRNA confirmed cytoplasmic localisation, whilst *SNORD3* expression is restricted to the nuclear compartment. Magnification X63 for all panels. (B) RNA-FISH of *LINC00961* in HUVECs (X63) Z-stacking slice to assess *LINC00961* localisation within the nucleus. (C) *LINC00961* qRT-PCR on cytoplasmic and nuclear fractions in HUVECs (n=8). *NEAT1* was used as a nuclear fraction control.

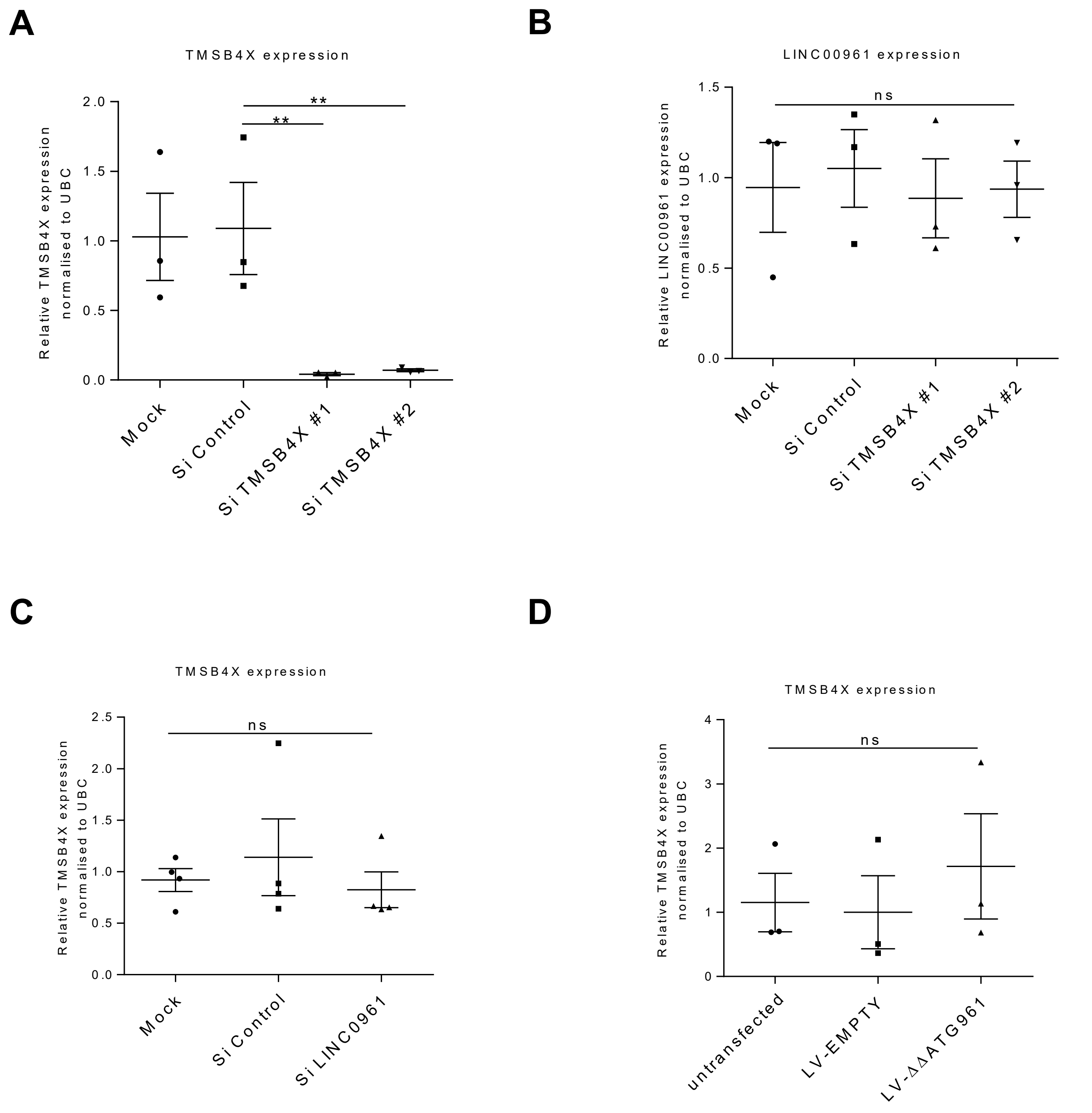

Supplementary Figure 9: ***LINC00961*** **does not regulate** ***TMSB4X*** **transcript level and** ***vice versa.*** (A) Confirmation of *TMSB4X* knockdown by qRT-PCR on dsiRNA treated HUVECs (compared to dsiRNA control and mock transfected cells) (n=3). (B) Impact of *TMSB4X* dsiRNA on *LINC00961* transcript expression as assessed by qRT-PCR (n=3). (C) Impact of *LINC00961* dsiRNA on *TMSB4X* transcript expression as assessed by qRT-PCR (n=4). (D) Impact of LV-ΔΔATG961 on *TMSB4X* transcript expression as assessed by qRT-PCR (n=3). Statistical analysis was done using unpaired t-tests. On the graphs, ** indicates $p<0.01$ and ns = not significant.

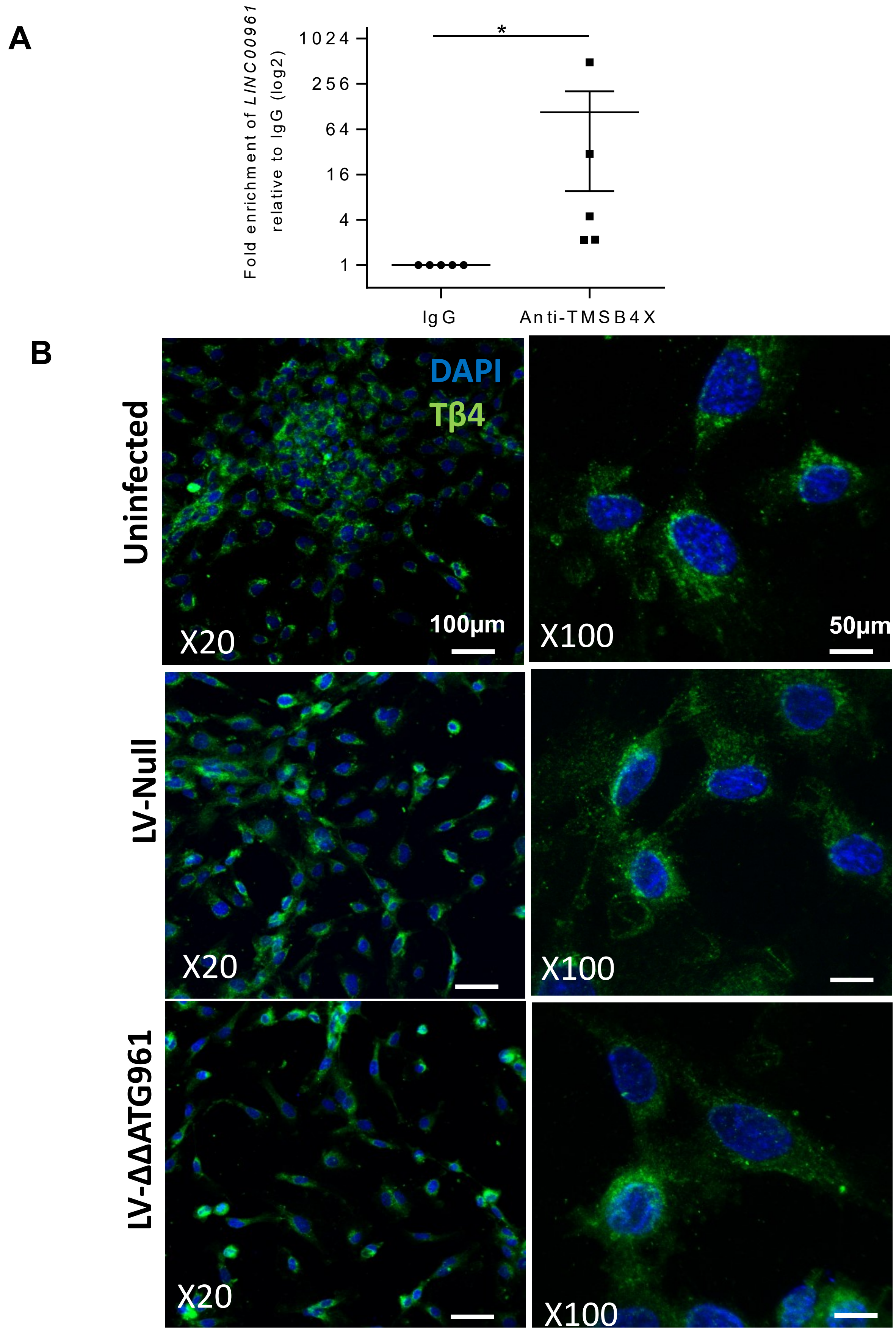

Supplementary Figure 10: Validation of thymosin beta 4-x and *LINC00961* interaction, and localisation of thymosin beta 4-x in HUVECs. (A) qRT-PCR analysis of *LINC00961* levels after pull-down with anti-Tβ4 antibody, student's t-test, * $p<0.05$, n=5. (B) Immunofluorescent staining of Tβ4 protein in HUVECs in uninfected, LV-null, and LV-ΔΔATG961 conditions. Tβ4 is green with nuclear DAPI in blue. Left hand panes show X20 magnification, scale bar 100μm, right hand panes show X100 magnification, scale bar 50μm.
